# Diubiquitin-Based NMR Analysis: Interactions Between Lys6-Linked diUb and UBA Domain of UBXN1

**DOI:** 10.3389/fchem.2019.00921

**Published:** 2020-01-22

**Authors:** Dharjath Shahul Hameed, Gabrielle B. A. van Tilburg, Remco Merkx, Dennis Flierman, Hans Wienk, Farid El Oualid, Kay Hofmann, Rolf Boelens, Huib Ovaa

**Affiliations:** ^1^Department of Cell Biology II, The Netherlands Cancer Institute, Amsterdam, Netherlands; ^2^Department of Cell and Chemical Biology, Oncode Institute, Leiden University Medical Centre, Leiden, Netherlands; ^3^NMR Spectroscopy, Bijvoet Center for Biomolecular Research, Utrecht University, Utrecht, Netherlands; ^4^UbiQ Bio BV, Amsterdam, Netherlands; ^5^Institute for Genetics, University of Cologne, Cologne, Germany

**Keywords:** isotope labeled diubiquitin, NMR, extended UBA domain, UBXN1, solid phase peptide synthesis

## Abstract

Ubiquitination is a process in which a protein is modified by the covalent attachment of the C-terminal carboxylic acid of ubiquitin (Ub) to the ε-amine of lysine or N-terminal methionine residue of a substrate protein or another Ub molecule. Each of the seven internal lysine residues and the N-terminal methionine residue of Ub can be linked to the C-terminus of another Ub moiety to form 8 distinct Ub linkages and the resulting differences in linkage types elicit different Ub signaling pathways. Cellular responses are triggered when proteins containing ubiquitin-binding domains (UBDs) recognize and bind to specific polyUb linkage types. To get more insight into the differences between polyUb chains, all of the seven lysine-linked di-ubiquitin molecules (diUbs) were prepared and used as a model to study their structural conformations in solution using NMR spectroscopy. We report the synthesis of diUb molecules, fully ^15^N-labeled on the distal (N-terminal) Ub moiety and revealed their structural orientation with respect to the proximal Ub. As expected, the diUb molecules exist in different conformations in solution, with multiple conformations known to exist for K6-, K48-, and K63-linked diUb molecules. These multiple conformations allow structural flexibility in binding with UBDs thereby inducing unique responses. One of the well-known but poorly understood UBD-Ub interaction is the recognition of K6 polyubiquitin by the ubiquitin-associated (UBA) domain of UBXN1 in the BRCA-mediated DNA repair pathway. Using our synthetic ^15^N-labeled diUbs, we establish here how a C-terminally extended UBA domain of UBXN1 confers specificity to K6 diUb while the non-extended version of the domain does not show any linkage preference. We show that the two distinct conformations of K6 diUb that exist in solution converge into a single conformation upon binding to this extended form of the UBA domain of the UBXN1 protein. It is likely that more of such extended UBA domains exist in nature and can contribute to linkage-specificity in Ub signaling. The isotopically labeled diUb compounds described here and the use of NMR to study their interactions with relevant partner molecules will help accelerate our understanding of Ub signaling pathways.

## Introduction

Ubiquitin (Ub) is a small protein of 76 amino acids, involved in the post-translational modification of several proteins in cells (Hochstrasser, 1996; Hershko and Ciechanover, 1998). Ub is attached to a target protein in a process called ubiquitination which employs a specific combination of three enzyme classes: Ub activating enzyme E1, Conjugating enzyme E2, and Ub ligase E3 (Scheffner et al., 1995). On the other hand, ubiquitin can be removed from its substrates by enzymes called deubiquitinases (DUBs) (Komander et al., 2009a). Ub is attached to a target protein as a monomer or as a polymeric chain (polyUb) in which individual Ub molecules are attached via their C-terminal residue to one of the seven lysine residues (K6, K11, K27, K29, K33, K48, and K63) or the N-terminal methionine residue of other Ub molecules (Meierhofer et al., 2008; Akutsu et al., 2016). Different types of Ub modifications cause different responses, such as regulation of protein turnover and DNA-repair signaling and are therefore ubiquitination is essential in maintaining cellular homoeostasis. The polyUb chains vary in length, type of linkage (homotypic or branched) and the position of the modified lysine residues in target proteins (Li and Ye, 2008). Recognition of different polyUb chains by Ub binding domains (UBDs) is essential for stimulation of Ub signaling pathways.

The enzymatic assembly of all but K27-linked homotypical ubiquitin chains can be achieved by using the required combination of ubiquitinating E1-E2-E3 enzymes (Zhang et al., 2005; Hospenthal et al., 2013; Michel et al., 2015; Faggiano et al., 2016). However, there is lack of control over the length of polyUb chains generated when using enzymatic methods and this often requires either mutating the Ub monomer to halt the chain extension or using extensive purification methods to separate different Ub polymers. In addition, such techniques are known for being less selective and require post-synthesis clean-up of undesired chains using chain-specific DUBs. This results in low yields and long preparation times. To circumvent this, in the past years, we and others have reported the synthesis of ubiquitin chains using chemical tools (El Oualid et al., 2010; Kumar et al., 2010; Moyal et al., 2012; van der Heden van Noort et al., 2017). The use of a thiolysine handle at the sites of ubiquitination and the omission of enzymes resulted in the generation of diUbs of all seven isopeptide linkages (Merkx et al., 2013). These chains have been used extensively to study the biochemical properties of DUBs (Faesen et al., 2011; Licchesi et al., 2011).

To study the structural behavior of diUb molecules in solution by nuclear magnetic resonance spectroscopy (NMR), segmental isotope-labeled diUb reagents can be a valuable tool. Such a diUb molecule consists of a labeled Ub moiety linked to an unlabeled Ub moiety at defined positions. Synthesis of labeled diUb molecules has been reported previously relying on expressing recombinant Ub using an evolved tRNA/tRNA-synthetase system, followed by selective deprotection, chemical ligation and purification of diUb molecules (Castañeda C. et al., 2011; Castañeda C. A. et al., 2011). These diUb molecules can be used to study the intermolecular interactions with other proteins involved in the ubiquitin pathway.

**Figure 1:**
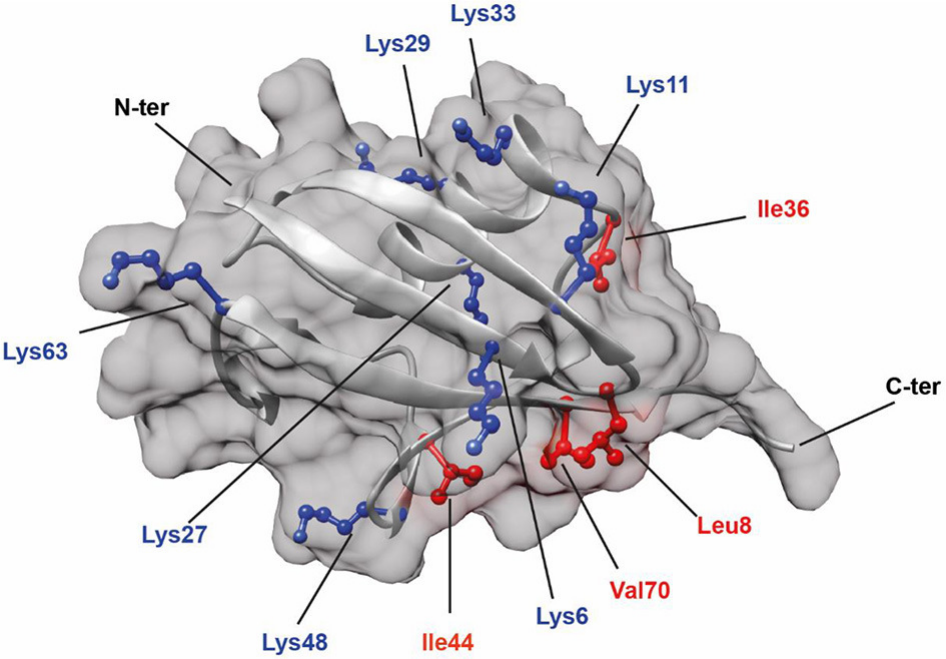
Structure of Ub (PDB: 1UBQ) showing the positions of all seven lysine residues (colored in blue). In addition, hydrophobic residues (colored in red) known to be involved in protein interactions are also highlighted.

It has been reported that Ub chain interactions with other proteins frequently involve a hydrophobic patch containing residues such as Leucine 8, Isoleucines 36 and 44, and Valine 70 on the ubiquitin surface (Figure 1, labeled in red) (Sloper-Mould et al., 2001). This patch is also involved in interactions between the Ub monomers in a diUb molecule or in polyUb chains. However, the position of interacting residues and the strength of the interaction between monomers differ for each Ub linkage (Wang et al., 2014). Although structural information on commercially available K48 (van Dijk et al., 2005; Ryabov and Fushman, 2007; Zhang et al., 2009) and K63 (Komander et al., 2009b; Weeks et al., 2009; Liu et al., 2015; He et al., 2016) Ub chains and other atypical Ub chains of K6- (Virdee et al., 2010; Hospenthal et al., 2013), K11- (Bremm et al., 2010; Matsumoto et al., 2010; Castañeda et al., 2013), K27- (Gao et al., 2016), K29- (Kristariyanto et al., 2015a), and K33- (Castañeda C. A. et al., 2011; Kristariyanto et al., 2015b; Michel et al., 2015) linkages is available, a comparative study on diUb structural dynamics in solution is necessary to get an idea on the differences in structure of different Ub linkages. Since structure-function relationships are known to be directive in ubiquitin signaling, it is essential to uncover the structural details of diUb molecules. For obtaining structural details, X-ray crystallography and increasingly also single-particle EM can be used to obtain high-resolution snapshots of protein folding and interactions of diUb molecules with some of their interacting proteins. On the other hand, NMR spectroscopy can provide a more dynamic view on structural transitions due to changes in environmental conditions and allows kinetic analyses of binding and dissociation between proteins and their interacting partners. In this study, we synthesized all seven isopeptide-linked diUbs using native chemical ligation of different proximal lysine-Ubs to a distal ^15^N-labeled Ub. A comparative study on the interactions between the ^15^N-labeled distal Ub and the unlabeled proximal Ub for each of the diUb linkages showed different interaction details in good agreement with previously reported data (Castañeda et al., 2016a,b). Furthermore, we demonstrate here the usefulness of these tools for gaining structural insights into the selective recognition of a unique Ub-binding domain (UBD) for a diUb linkage.

Each ubiquitin linkage-type leads to a different response in cells, based on their recognition by specific proteins containing a UBD. UBDs provide a structural basis for different responses by recruiting Ub chains and other proteins associated in their respective pathway. For example, the DNA repair pathway is one of the crucial pathways in cells that utilize polyUb signaling and is essential in maintaining genomic integrity during or after cell division. DNA damage can be repaired by several mechanisms (Schwertman et al., 2016). Among them, Non-Homologous End Joining (NHEJ) and Homologous Recombination (HR) are the most prevalent DNA-damage repair pathways. It has been observed that a Ubiquitin ligase called BRCA1 is involved in both of these DNA repair pathways. BRCA1 is an oncogene that is mainly associated with the prevalence of breast cancer (Rosen et al., 2003).

The BRCA-mediated DNA repair pathway involves the recognition of K6 polyubiquitin chains on BRCA1 protein by another protein called UBXN1 (Ohta et al., 2011). The UBXN1 protein contains a UBD that belongs to the family of ubiquitin-associated domain (UBA) at its N-terminal tail (Wu-Baer et al., 2010). The UBA domain is one of the earliest types of defined ubiquitin-binding domains described in literature (Hofmann and Bucher, 1996). These domains are short (about 45 amino acids) polypeptide sequences and are frequently observed in the enzymes associated with the ubiquitin machinery. The UBA domains usually consists of three alpha-helix modules which include a highly conserved hydrophobic surface that can bind efficiently with hydrophobic areas of Ub or polyUb chains (Mueller and Feigon, 2002). The UBA sequences are conserved among proteins and enzymes involved in the proteasome degradation pathway (Chen et al., 2001) and in DNA repair (Kozlov et al., 2007).

Although it has been established that the UBA domain of UBXN1 can specifically recognize a K6 polyUb chain attached to the BRCA1 Ub ligase (Wu-Baer et al., 2010), the mode of interaction between the isolated UBA domain and the K6-Ub chain is largely unknown. Using our synthetic diUbs and biophysical techniques, we established how only an extended version of the UBA domain (UBAext1-52) of the UBXN1 protein binds selectively to K6 diUb. To illustrate the interaction of K6 diUb with UBAext1-52 of the UBXN1 protein, we monitored their titration by NMR and revealed which residues in the distal Ub of the K6 diUb molecule are important for this interaction. Understanding this interaction between the extended UBA domain and K6 Ub chains will help in understanding the interaction preference over other Ub chains.

## Materials and Methods

### Expression of UBE1 Enzyme and ^15^N Isotopic Labeling of Ubiquitin

All chemicals were obtained from Sigma unless stated otherwise. The ubiquitin-activating enzyme (UBA1) was recombinantly expressed with N-terminally fused hexahistidine tag (His6-tag). The enzyme was expressed in BL21 *E.coli* cells by adding 1 mM IPTG when the OD600 reached 0.6, followed by culturing the cells at 18°C overnight. Cells were then sonicated in a lysis buffer containing 20 mM Tris-HCl, 250 mM NaCl and 5 mM 2-Mercaptoethanol at pH 8. The supernatant was incubated with TALON® metal affinity resin and after two washing steps, the UBA1 was eluted at 250 mM Imidazole concentration in the elution buffer. The imidazole was removed from the buffer using 10 kDa cut-off spin columns (Millipore). The final concentration of the enzyme was measured using a Nanodrop™.

^15^N-enriched ubiquitin was expressed as an untagged protein using a pET2A expression system in BL21 *E.coli* cells in minimal essential medium. The M9 minimal essential medium contained 50 mM Na_2_HPO_4_, 50 mM KH_2_PO_4_, 5 mM Na_2_SO_4_, 50 mM ^15^NH_4_Cl, 2 mM MgSO_4_, 0.01% glycerol, 0.001% glucose, and 0.004% lactose (inducer). After expression by autoinduction at 37°C overnight, cells were spun down at 3,700 G for 10 min and resuspended in Milli-Q™ water containing protease inhibitor cocktail tablets. Then the suspension was heated to 85°C for 30 min, cooled down to room temperature and added with 0.3 mg DNase per 50 mL suspension along with 10 mM MgSO_4_. After heating again at 85°C for 30 min, the cell lysate was spun down at 20,000 rcf. The supernatant was purified by cation-exchange chromatography at 4°C using AKTA Unichromat 1500- “PRO” system (15 × 185 mm column packed with Workbeads™ 40 S) with two mobile phases: 50 mM NaOAc, pH 4.5 (solvent A), and 1 M NaCl in 50 mM NaOAc (solvent B), pH 4.5 (Flow-rate 5 mL/min). All fractions were checked on an SDS-PAGE gel. The pure fractions collected from the cation-exchange column were re-purified over a C18 Atlantis preparative reverse-phase HPLC on a Shimadzu Prominence system using two mobile phases: A = 0.05% TFA in water and B = 0.05% TFA in CH_3_CN (Column temperature 40°C, flow rate 7.5 mL/min, UV-signal is measured at 230 and 254 nm). Typical ubiquitin yields were 80 mg/L of cell culture.

### Preparation of Lysine-Linked Diubiquitin Molecules

The ^15^N-Ub-MESNa thioester was obtained according to a previously reported procedure with >95% yield, which was then purified using RP-HPLC and lyophilized (Oualid et al., 2012). ^15^N-Ub-MESNa thioester ligations were performed using the following conditions: 125 mM HEPES-NaOH pH 8; 100 mM MESNa; 10 mM MgCl_2_; 10 mM ATP and 250 nM UBA1 enzyme at a concentration of 550 μM ^15^N Ubiquitin. The ^15^N-Ub-MESNa thioester was then purified using reversed-phase HPLC (RP-HPLC). Ub (K6, K11, K27, K29, K33, K48, and K63) δ-thiolysine derivatives were prepared using chemical synthesis on a solid phase. Diubiquitins were synthesized using a previously reported procedure (El Oualid et al., 2010). Native chemical ligation was performed by adding equal amounts of ^15^N Ub MESNa thioester and thiolysine-Ub to a final concentration of 50 mg/mL in 6 M Gnd.HCl 0.2 M sodium phosphate buffer pH 8 containing 100 mM MPAA and 50 mM TCEP. After overnight ligation, the product was analyzed by LCMS and then diluted in desulphurization mix to a final concentration of 1 mg/ml protein (Diubiquitin). This mix contains 6 M Gnd.HCl 0.2 M sodium phosphate buffer pH 6.8, 200 mM TCEP, 50 mM reduced Glutathione, and 50 mM radical initiator VA-044 (2,2'-Azobis[2-(2-imidazolin-2-yl)propane]dihydrochloride). After overnight desulphurization, the product was analyzed by LCMS and purified with RP-HPLC.

### Preparation of UBA Peptides

UBA(1-42) and UBA(ext1-52) peptides were synthesized at 2 μmol scales, coupled with TAMRA on the N-terminus and purified by reversed-phase HPLC. Stock concentrations of TAMRA-UBA peptides were measured using a standard curve of TAMRA-K-G from 0 to 800 nM in 20 mM Tris pH 7.6 and 150 mM NaCl.

The amino acid sequence of the UBA domain of the UBXN1 protein is as follows:


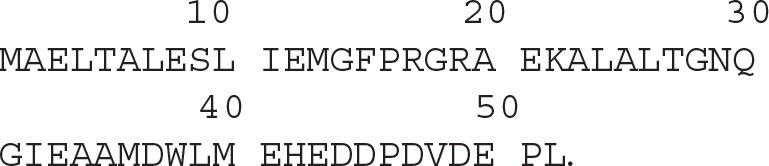


### Analysis of Ubiquitin and Diubiquitin Molecules

The Ub and diUb molecules were analyzed by 12% Nu-PAGE SDS gel electrophoresis using MES buffer and Seablue plus 2® as a protein marker. Isolated products with an expected molecular weight (MW) of 17,212 Da were observed as a single band in the gel at around 17 kDa. The MW of the product were also confirmed by LC/MS using a Phenomenex Kinetex C18 (2.1 × 50 mm, 2.6 μm) column (flow rate: 0.8 mL/min; runtime: 6 min; mobile phases: A = 1% CH_3_CN, 0.1% formic acid in water and B = 1% water and 0.1% formic acid in CH_3_CN; column T = 40°C. Protocol: 0–0.5 min: 5% B; 0.5–4 min: 5–95% B gradient; 4–5.5 min: 95% B). Final yields were measured after freeze-drying the product.

For Circular Dichroism (CD) measurements, a JASCO CD J1000 machine was used (UMC, Utrecht, the Netherlands). Samples were dissolved in DMSO and then diluted in NMR buffer containing 20 mM NaH_2_PO_4_ pH 6.8 to a final concentration of 4 μM. Measurements were performed at 25°C using wavelengths ranging from 260 to 185 nm in a span of 100 m deg. The scanning speed was 20 nm/min and measurements from 10 experiments were averaged. After CD measurements, the samples were subjected to BCA assay to determine actual concentrations. Based on the observed values of CD measurements and concentration from BCA assay, CD plots were prepared.

### NMR Measurements

Freeze-dried ubiquitin and diubiquitin samples were dissolved in 5% DMSO (Biosolve) in Milli-Q® water and then redissolved in NMR buffer containing 20 mM NaPO_4_ pH 6.8 and 10% D_2_O. Then, samples were taken in 15 ml 3.5 kDa Millipore spin filter tubes and spun-washed with three volumes of NMR buffer until DMSO was almost completely removed (LC/MS analysis). Concentrated samples were diluted to 500 μL with NMR buffer and the final concentration was determined using BCA assay using ubiquitin as standard. The pH was carefully measured using a Mettler TOLEDO pH probe.

All NMR studies were carried out on a Bruker 900 MHz spectrometer with a TCI cryoprobe, at 298 K (25°C). [^1^H, ^15^N] HSQC-spectra were acquired, processed and calibrated using standard methods. Chemical Shift Perturbations (CSPs) were calculated by comparing the [^1^H, ^15^N] HSQC spectra of mono Ub with that of each of the diUb molecules/ The CSP was calculated according to the following formula

$$CSP=\sqrt{{\left(0.2\Delta \delta \text{N}\right)}^{2}\;+\;{\left(\Delta \delta \text{H}\right)}^{2}}$$

where ΔδH and ΔδN are the chemical shift differences for ^1^H and ^15^N, respectively.

The spectra of K6 diUb indicated two different co-existing conformations. An “open conformation” was assigned based on similarity with the mono-Ub spectrum.

### Fluorescence Polarization and Microscale Thermophoresis Measurements

Fluorescence polarization (FP) measurements were performed at room temperature preceded by overnight incubation of UBA(ext1-52) domain with diubiquitin at 4°C. Total assay volume was 20 μL in black 384-well plates (low volume, flat bottom, non-binding surface; Corning® ref 3820). All diubiquitin variants and concentrations were measured in triplicate. The concentration of synthetic TMR-labeled UBA domain was unchanged at 5 nM while diubiquitin was added in six steps of increasing concentrations from 0.78 to 25 μM. A UBA domain-only control (0 μM diubiquitin) was used to normalize measured FP values to 0. For these measurements, native diubiquitins were used and prepared as described previously (El Oualid et al., 2010). DiUbs were additionally purified by gel filtration on a HiLoad 16/600 superdex 75 pg column (GE Healthcare) in 20 mM Tris pH 7.6 and 150 mM NaCl. The measurements were carried out in a FP binding buffer (20 mM Tris pH 7.6, 150 mM NaCl, 0.5 mg/ml BGG, 1% TX-100). Before each measurement, the plates were briefly centrifuged for 1 min at 4°C and 500 G. Read-out was performed on a PHERAstar plate reader (BMG labtech) using a TAMRA filter. Statistical analyses were performed with GraphPad Prism 7 software using non-linear regression analysis [one site binding (hyperbola)].

Microscale thermophoresis (MST) measurements were carried out using the synthetic TAMRA-UBAdomains in FP binding buffer. Concentrations of K6 diUb ranged from 1.53 to 50 μM. Samples were incubated for 30 min to allow binding and measured in hydrophobic capillaries on a Monolith NT.115 reader (NanoTemper Technologies, Munich, Germany) using 30% LED and 40% IR-laser power. The analysis was performed with GraphPad Prism 7 software using non-linear regression analysis [log (inhibitor) vs. response (three parameters)].

## Results

### diUb Synthesis and Validation by Gel, LCMS

**Figure 2:**
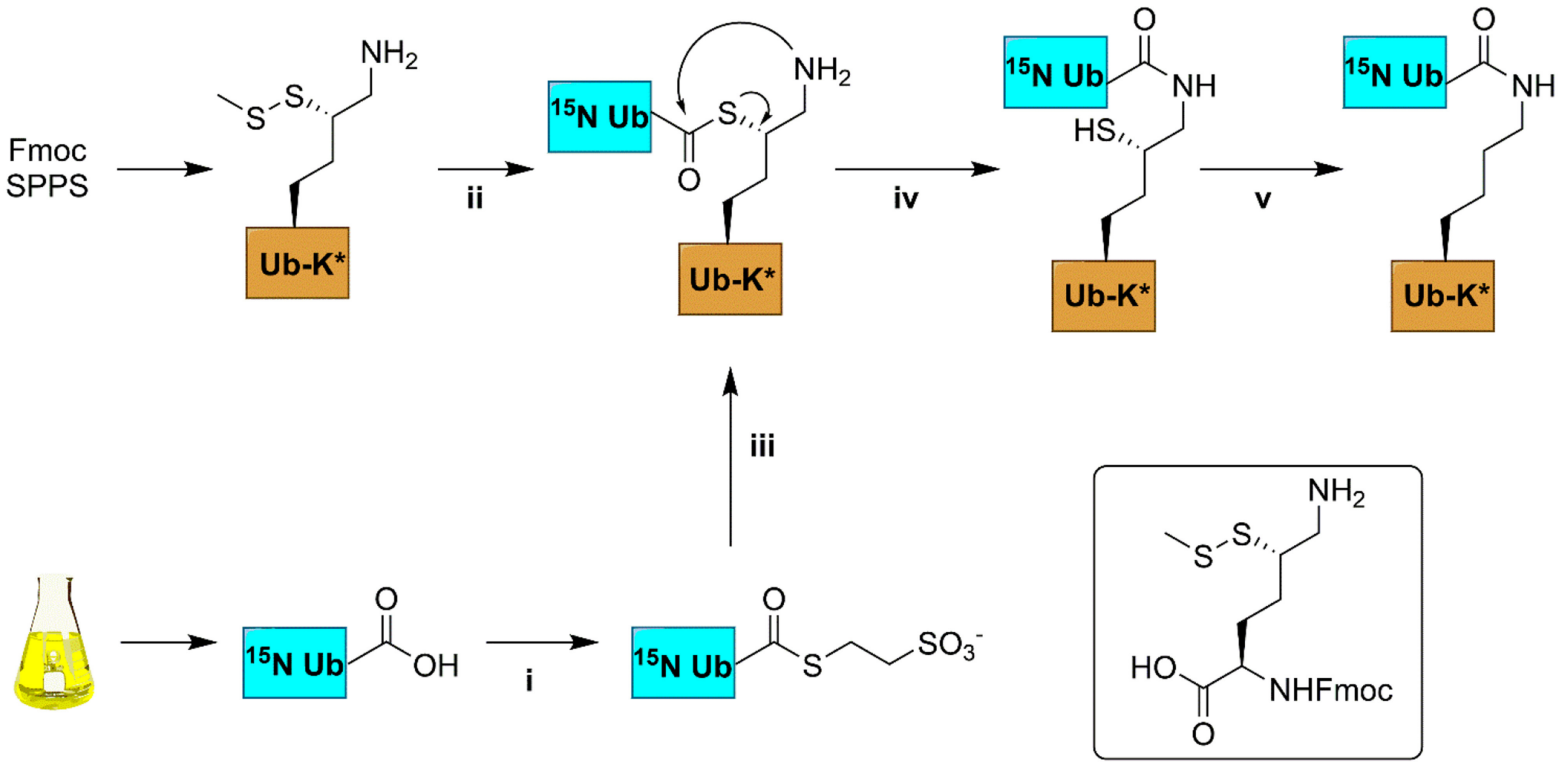
Schematic representation of the synthesis of ^15^N-labeled diUb. ^15^N-Ub was expressed in a bacterial expression system; thiolysine (inset) containing Ub was synthesized from Fmoc-based SPPS. (i) 100 nM UbE1, 100 mM MESNa, pH 8; (ii) 50 mM TCEP, 6 M Gnd.HCl; (iii) Ub-thiolysine after step (ii), 100 mM MPAA, 6M Gnd.HCl, pH 8; (iv) room temperature, overnight incubation; (v) buffer exchange to remove MPAA, 100 mM TCEP, 100 mM VA-044, 6 M Gnd.HCl, pH 7.

Diubiquitin molecules were synthesized using our previously established native chemical ligation procedure (Figure 2) (El Oualid et al., 2010). Briefly, the different proximal Ub moieties, containing a δ-thiolysine building block instead of a lysine residue, were generated using Fmoc SPPS. The distal ^15^N-Ub part was prepared by recombinant bacterial expression in ^15^N-ammonia enriched M9 minimal medium and converted to ^15^N-Ub MESNa thioester using UbE1 enzyme and MESNa. The proximal and ^15^N-distal Ub precursors were ligated using native chemical ligation conditions. The product was then subjected to chemical desulfurization using TCEP and VA-044 and finally purified by reversed-phase HPLC.

The purified product was dissolved in DMSO and refolded into NMR buffer (20 mM NaPO_4_ pH 6.8 and 10% D_2_O). ^15^N-Ub was also purified by HPLC and refolded using the same procedure. To check for proper folding, the products were examined by Circular Dichroism (CD) using commercially available Ub as a control. Based on SDS-PAGE analysis (Supplementary Figure S1A), the CD spectra (Supplementary Figure S1B) and LC/MS analysis, the distal ^15^N labeled diUbs (Supplementary Figures S14–S21) are found to be pure and properly refolded.

### Comparison of NMR Data of Monoub and diUb Molecules

By NMR, a 2D [^1^H.^15^N] HSQC spectrum was obtained for ^15^N-Ub (Supplementary Figure S2). Although most of the signals were identified and assigned according to a previously reported data (Cornilescu et al., 1998), signals corresponding to Met1, Glu24, and Gly53 backbone amides were missing. The data showed that monoUb is properly folded.

**Figure 3:**
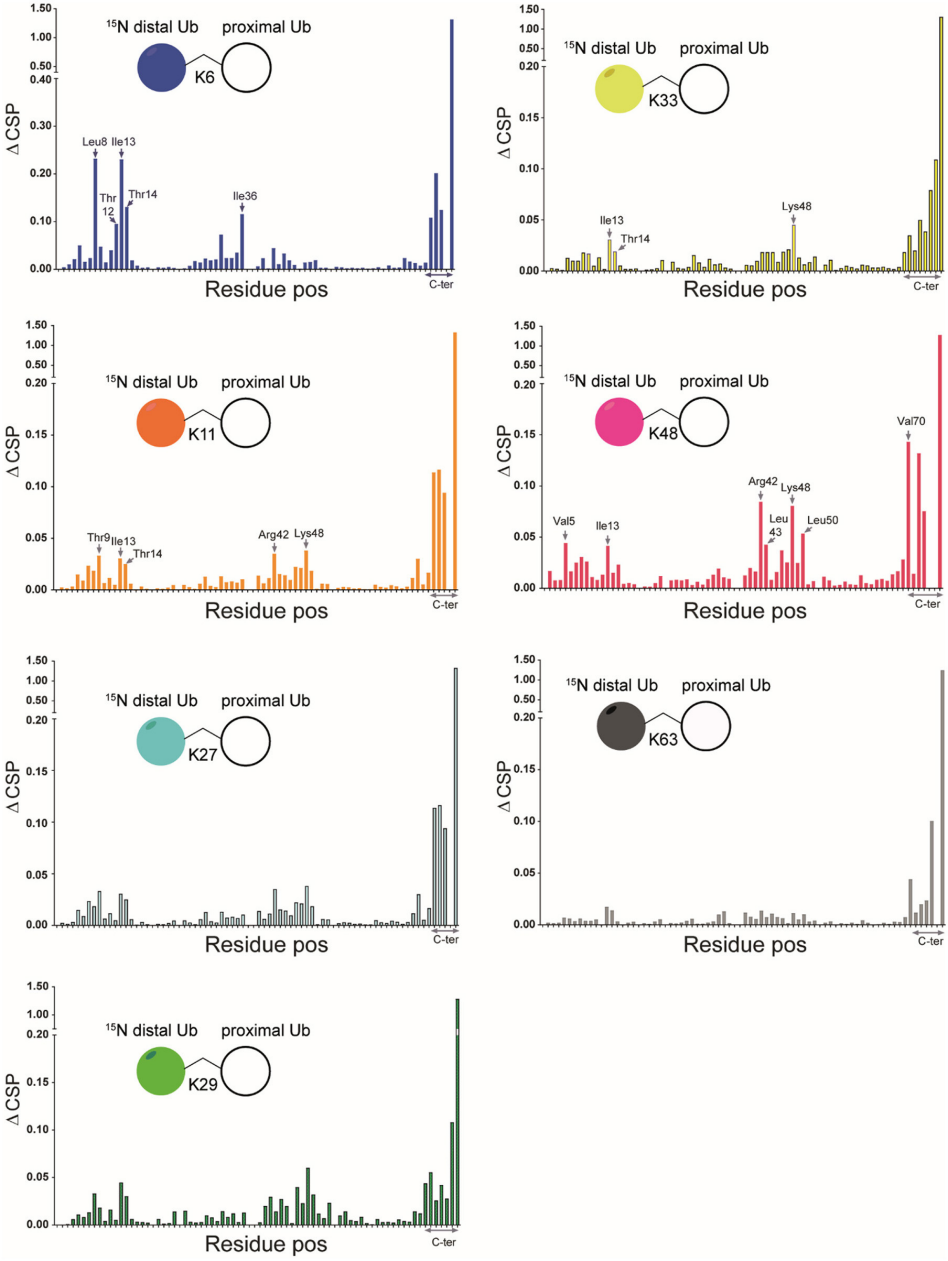
CSPs calculated for all isopeptide linked diUbs by comparison of ^15^N-^1^H HSQC spectrum of mono-Ub with that of each of the ^15^N-labeled diUb. Pictorial representations of each of the diUbs are shown (in each panel). In general, the C-terminal residues in all diUbs show CSP due to their covalent bonding with the second unlabeled Ub. However, other residues also show changes, indicating their possible interaction with the unlabeled proximal Ub. The residues that show major CSP besides the C-terminal region are labeled.

We compared the [^1^H, ^15^N] HSQC spectra of each of the different ^15^N-diUb molecules (Supplementary Figures S3–S9) (hereafter referred to as diUbs) to that of monomeric ^15^N-Ub to reveal interactions between the distal Ub and proximal Ub moieties. Chemical shift perturbations (CSP) were calculated from ^1^H to ^15^N resonance frequency-differences between signals of the same residue in both monoUb and diUb spectra. This was plotted in a graph, illustrating the influence of the attached proximal Ub on residues in the ^15^N-distal Ub moiety (Figure 3). Previously using a similar approach, the K48 (van Dijk et al., 2005; Hirano et al., 2011; Lai et al., 2012) and K63 (Jacobson et al., 2009; Liu et al., 2015) diUbs have been extensively studied. In our experiments, we also analyzed the NMR spectrum of all other diUb molecules.

CSPs are useful in determining the changes in the local environment of amino acids, which can be attributed to direct or indirect interactions but cannot be differentiated as such. All diUb spectra showed a common CSP behavior in the C-terminal region of the distal Ub module, where the isopeptide linkage with the proximal Ub module is located. However, the hydrophobic region in Ub including the residues of Leu8, Ile36, Ile44, and Val70 and its surroundings also showed CSPs to a varying degree of magnitude and signal shift directions. In the case of K6 diUb, spectral changes were mostly observed for Leu8, Ile36, and a small region in the second beta-sheet covering residues Thr12, Ile13, and Thr14. K11 diUb showed similar behavior encompassing residues Thr9, Ile13, Thr14, and Arg42. Here, Lys48, which is in the hydrophobic region surrounding Ile44 residue, was also disturbed. The elusive K27 diUb showed changes for Thr9 and Lys48 nearby the hydrophobic patch that surrounds Leu8 and Ile44 residues, respectively. K29 diUb showed disturbances in Leu8, Ile13, Thr14, and Lys48, similar to that of K11 diUb. Intriguingly, the spectra of K27 diUb and K29 diUb show variation likely because the lys29 residue in K29 diUb is more solvent-exposed compared to lys27 in K27 diUb. Similar effects as with K29 diUb were also observed for K33 diUb. K48 diUb, which is the most studied so far, showed CSPs for Val5, Ile13, areas around Ile44 and Val70, encompassing the hydrophobic patch of Ub, suggesting a compact folding as had been observed in X-ray crystal structures of K48 polyUb chains (Varadan et al., 2005). Finally, K63 diUb shows the least interactions between the distal Ub and proximal Ub, in line with the reported open conformations known for K63 linked Ub chains. Comparing the overall CSPs of each of the diUbs measured in our NMR experiment, we observed that K6 diUb, K11 diUb, K29 diUb, and K48 diUb showed more perturbations than K27 diUb, K33 diUb, and K63 diUb.

**Figure 4:**
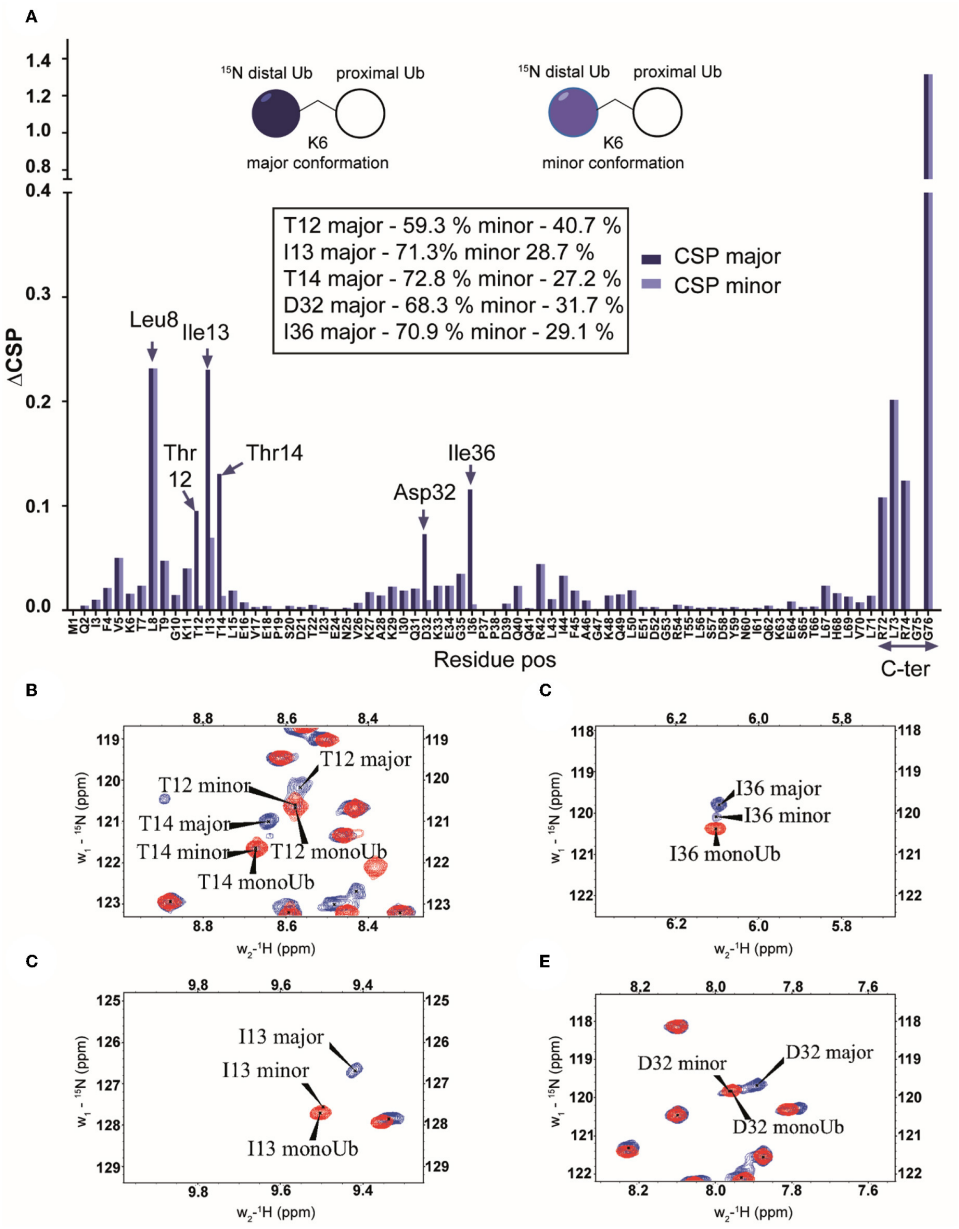
**(A)** Chemical shift perturbations calculated by comparing the ^15^N-^1^H spectrum of mono-Ub and the distally ^15^N-labeled K6-diUb (structural representation in inset). Although most of the signals are less affected, certain residues like Leu8, Thr12-Thr14, Ile36, and the C-terminal tail from Arg72 to Gly76 are all shifted significantly. This indicates a change in the electronic environment of these residues, which may be attributed to interactions with the unlabeled proximal-Ub. Leu8 and Ile36 show a considerable migration relative to other residues. In addition, signal doubling is observed for Asp32 and Ile36 in K6-diUb. **(B–E)** NMR spectral regions showing ^15^N-^1^H peaks of Thr12, Ile13, Thr14, Asp32, and Ile36 of K6 diUb (blue) compared with monoUb (red).

Of particular interest was the K6 diUb spectrum which showed signal-doubling for Thr12, Ile13, and Thr14 and residues Asp32 and Ile36 (Figures 4A–E). After ruling out the presence of impurities in the K6 diUb sample (Supplementary Figures S1A, S15), we further analyzed this phenomenon. Based on the reported crystal structure for K6 diUb, the region around Asp32 and Ile36 is away from the interface between the two Ub moieties (Virdee et al., 2010). Our data suggest that there is a second conformation in solution. Assuming that relaxation properties and NMR lineshapes between the two conformations are similar we estimate the major and minor populations in an approximate ratio of 70:30 for K6 diUb (Figure 4). In the major conformation, Leu8, Asp32, and Ile36 could interact with Thr12, Ile13, and Thr14 residues (“loop-in” conformation) which is in agreement with a compact diUb fold. In the minor conformation, there is less effect from Ile36 and therefore less perturbations are seen in Thr12, Ile13, and Thr14 residues (“loop-out” conformation) indicating that this K6 diUb conformation is less compact than the closed one but comparable to K48 diUb.

### A Novel C-Terminally Extended UBA Domain of the UBXN1 Protein Binds Specifically to K6-Linked Diubiquitin *in vitro*

K6-linked polyubiquitin chains are known to be involved in BRCA-mediated DNA repair (Ohta et al., 2011). The BRCA1 protein forms a complex with BARD1 to gain its ubiquitin ligating activity. In addition to ubiquitinating many substrates involved in the DNA repair pathway with K6-linked polyUb chains (Sato et al., 2008), the BRCA1-BARD1 heterodimer complex can also auto-ubiquitinate itself with K6-linked polyUb chains (Chen et al., 2002; Wu-Baer et al., 2010). In this auto-ubiquitinated state, BRCA1-BARD1 ligase activity is significantly reduced by binding to the protein UBXN1 (Wu-Baer et al., 2010). UBXN1 contains an N-terminal UBA domain (residues 1–42) that binds to K6-linked polyubiquitin chains conjugated to BRCA1, while the C-terminal sequences of UBXN1 bind the BRCA1/BARD1 heterodimer in a ubiquitin-independent fashion (Wu-Baer et al., 2010). However, the isolated UBA(1-42) domain of UBXN1 did not bind with K6 polyUb chains, while deletion of this section in full length protein did abolish K6 interaction. This implied to us that there might be more residues beyond the UBA domain that are important for the K6-linked ubiquitin interaction (Wu-Baer et al., 2010).

**Figure 5:**
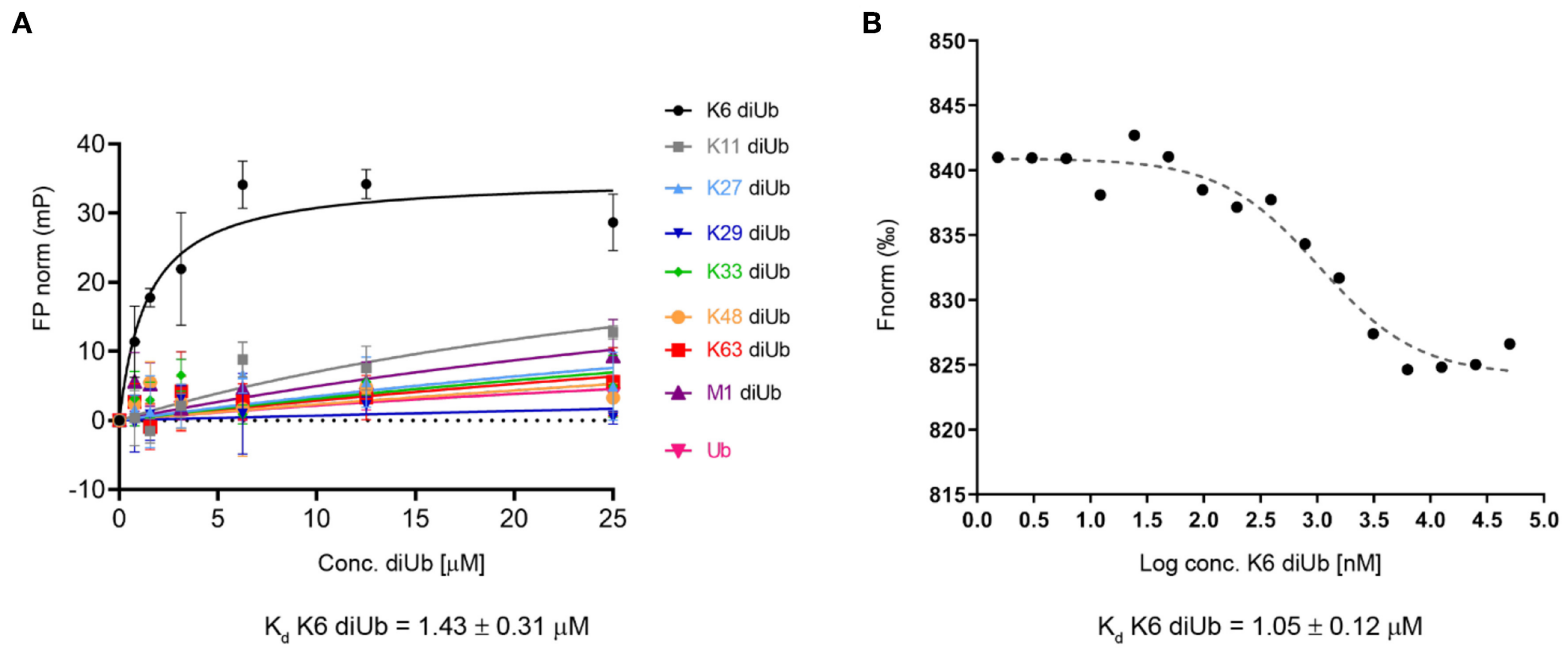
**(A)** Fluorescence polarization assay using a TAMRA-labeled UBXN1 UBA(ext1-52) domain and different concentrations of all 8 homotypical diUbs and monoUb. **(B)** Microscale thermophoresis binding curve of K6 diUb to TAMRA-labeled UBA(ext1-52) from UBXN1. These experiments show the preference and tight binding of UBA(ext1-52) to K6 diUb.

To study this in more detail, we set out to investigate the specificity of the UBXN1 UBA domain for K6 diUb molecules using a Fluorescence Polarization (FP) binding assay in which TAMRA-labeled UBA peptide was added to different concentrations of unlabeled diUbs of all linkage types. Consistent with the findings of Wu-Baer et al., we also did not observe binding of K6 diubiquitin with the canonical UBA domain (1-42) of UBXN1 (Supplementary Figure S10) (Wu-Baer et al., 2010). On comparing the UBA domains of other proteins, we found that the 10 amino acids following the C-terminus of all conventional UBA domains that we compared showed the existence of a conserved sequence (Table 1). Interestingly when looking at the alignment, a previously unnoticed WxxxH motif was found to be conserved only in the extended versions of the UBA domain and not the shorter ones. To investigate whether this C-terminally extended version of the UBA domain of UBXN1 had any effect on binding to K6 diUb, we repeated the FP binding assay with the UBA (1-52) domain. We observed a tight and linkage specific binding to K6 diubiquitin (Figure 5). We quantified the linkage specific binding of UBA(ext1-52) to K6 diUb with an approximate K_d_ of 1.43 ± 0.31 μM which was validated with an orthogonal technique called microscale thermophoresis (MST) and found a similar K_d_ value of 1.05 ± 0.12 μM.

**Table 1:**
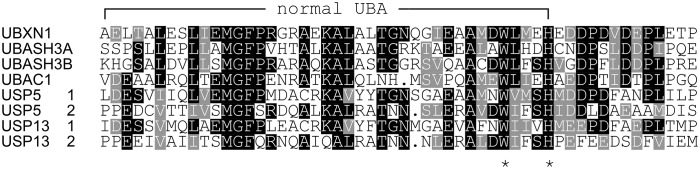
Comparison of UBA domain sequences from different Ub binding proteins.

The C-terminal extension adds about 10 amino acids at the C-terminal end of the conventional UBA domain. Moreover, all extended UBA domains have a totally invariant WxxxH motif within the 3rd helix. While this region is part of the conventional UBA fold, the conservation of this motif is only found in extended UBA-domain-containing members. The Trp and His residue in the conserved WxxxH motif is indicated with a star (^*^).

**Figure 6:**
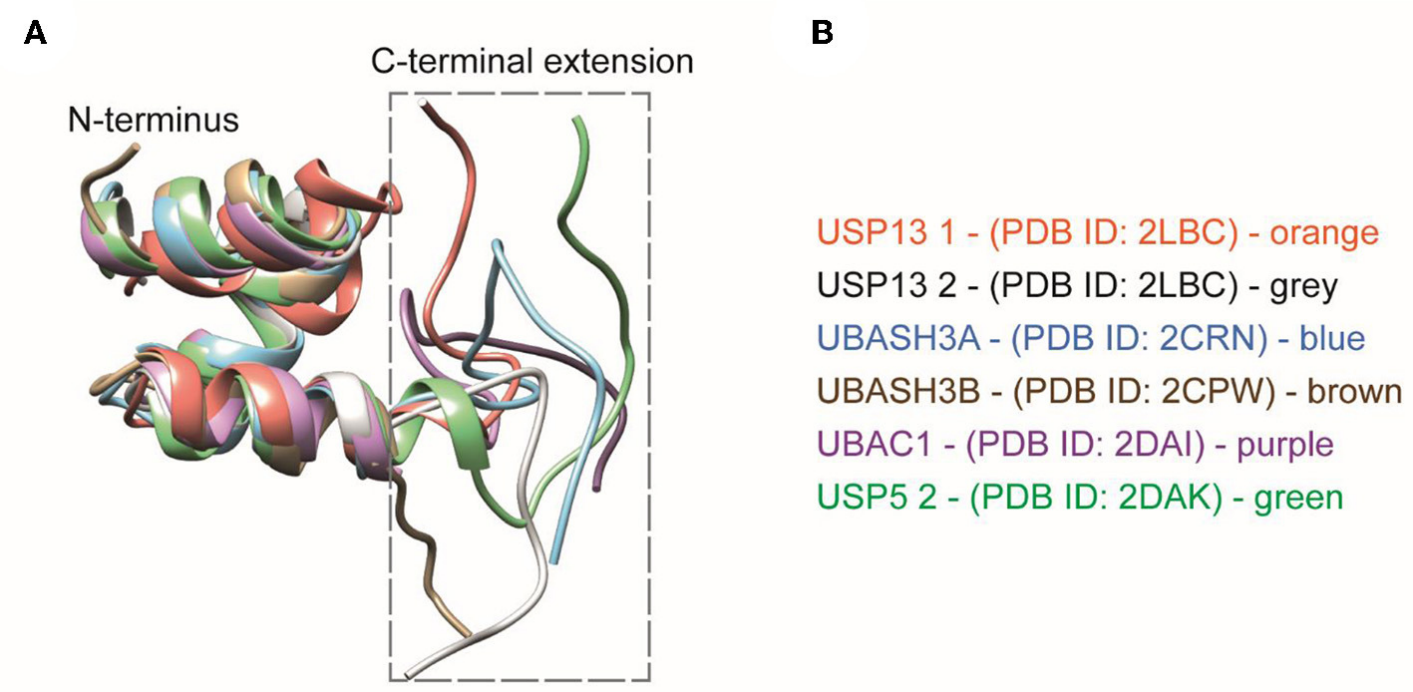
**(A)** Structural comparisons of extended UBA domains. The C-terminal extension of all the UBA domains mentioned here is found to be disordered. **(B)** The UBA-domain containing proteins are color coded along with their respective PDB IDs.

Carefully analyzing the NMR structures of the isolated UBA domains of UBASH3A (pdb: 2CRN), UBASH3B (pdb: 2CPW), UBAC1 (pdb: 2DAI), USP5 UBA2 (pdb: 2DAK), and USP13 (pdb: 2LBC), we found that all three alpha-helices in the conventional UBA domain are structurally conserved whereas the first few residues of the 10 residues extending from the C-terminus starts from the last alpha-helix and then becomes largely unstructured (Figure 6). The C-terminal UBA extension in UBXN1 seemingly adds to K6 diubiquitin specificity and further research is needed to investigate whether this holds true for the other proteins containing this conserved C-terminal UBA extension and thereby establishing a functional role of this conserved motif.

### NMR of K6 diUb With the UBA (1-52) Domain of UBXN1 Provides an Insight Into the Mode of Interaction

**Figure 7:**
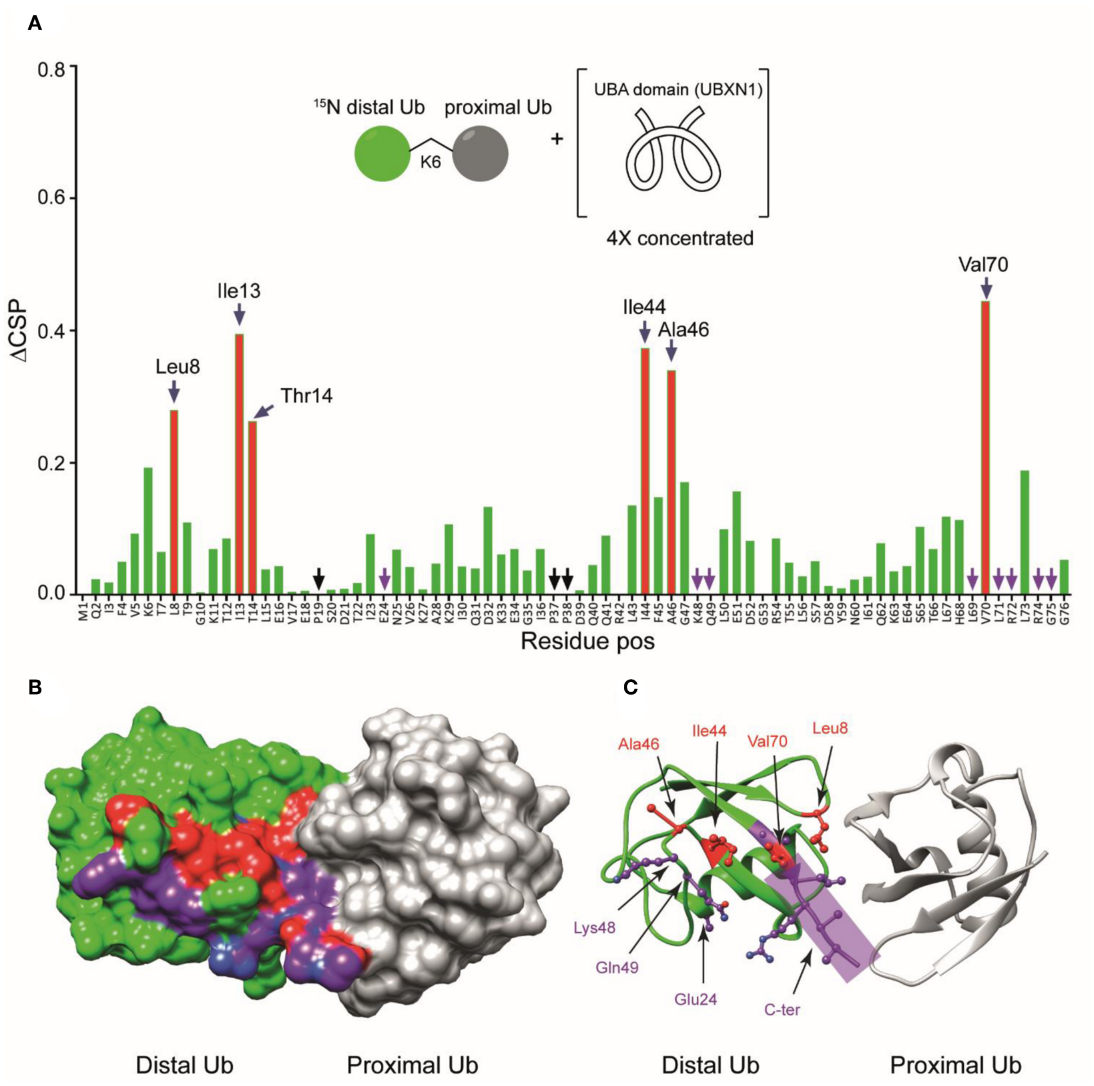
**(A)** Unlabeled UBA(ext1-52) domain of UBXN1 was added in different concentrations to ^15^N-K6 diUb and the CSPs were monitored. At a ratio of 4:1 (UBA(ext1–52) domain:K6 diUb), residues Leu8, Ile44, Ala46, and Val70 (red bars, labeled) shifted more than the rest. Other residues like Tyr 59 remain unchanged. **(B)** X-ray crystal structure of a K6 diUb (PDB: 2XEW) showing the residues that were perturbed according to CSP. Residues that shifted more are colored in red. Residues whose signal disappeared upon addition of UBA(ext1-52) peptide are represented in purple. **(C)** The same structure in figure **(B)** but showing the positions of side chains of the residues that were affected upon UBA(ext1-52) binding. Several perturbed residues are found to be positioned on the surface away from proximal Ub.

To further study the interaction between the UBA(ext1-52) domain of UBXN1 and K6 diUb, we titrated the UBA(ext1-52) with ^15^N-K6 diUb and monitored this by NMR. Signals corresponding to Lys 48, Gln49, Leu69, Leu71, and Leu73 disappeared after adding more than 1 equivalent of UBA(ext1-52), suggesting that these sites are in direct interaction with the UBA peptide. For other residues, signal shifts were observed. The CSP results indicated a distinct role of the hydrophobic patch on the distal Ub moiety that encompasses the residues Leu8, Ile44, Ala46, and Val70. Moreover, the residues Val5 to Thr9, Lys11, Ile13, and Thr14, surrounding Leu8 of the distal Ub, were also perturbed (Figure 7A, Supplementary Figure S11). Interestingly, shifts in Thr12, Ile13, and Thr14 were observed and explained previously as the “loop-in” and “loop-out” conformations for K6 diUb (Hospenthal et al., 2013).

Some signals that were split in the reference spectrum converged upon the addition of UBA(ext1-52) peptide. For example, Thr12, Ile13, and Thr14 were split in the unbound K6 diUb spectrum, but upon adding increasing concentrations of the UBA(ext1-52) peptide, their signals converged (Supplementary Figure S12). This indicates that the two different conformations of K6 diUb change into a single conformation upon binding with UBA(ext1-52) peptide. The fact that Ile44 and Leu8 show higher CSP values implying that the K6 diUb molecule is changing preferring the “loop-out” conformation upon interacting with the UBA peptide. However, residues Asp32 and Ile36 (Supplementary Figure S13) remained doubled, suggesting that the binding to the UBA(ext1-52) domain has local effects, but does not affect the structure of the entire distal Ub module.

Using the known X-ray crystal structure of K6 diUb, the interacting residues were mapped on the Ub surface (Figures 7B,C). It appears that the residues interacting with the extended UBA peptide are positioned away from the proximal Ub moiety. The fact that the Leu8 residue of distal Ub is positioned at the interface between the distal Ub and proximal Ub moieties may suggest a dual role for this residue in interacting with both the proximal Ub and UBA peptide.

## Discussion

Structures of all seven isopeptide-linked diUb molecules have been characterized using X-ray crystallography (Weeks et al., 2009; Bremm et al., 2010; Virdee et al., 2010; Hirano et al., 2011; Kristariyanto et al., 2015a,b; Pan et al., 2016). These crystal structures broadly fall into two categories: compact (K6, K48, K11, K27, K29, K33) and open (M1, K63) conformations (Wang et al., 2014). Some Ub chains, however, are known to exist in intermediate forms in solution. For example, K48 chains obtain two different conformations in addition to several intermediate structures (Lai et al., 2012). This structural flexibility is essential to facilitate polyUb signaling where K48 polyUb chains contribute to proteasomal degradation (Jacobson et al., 2009). Although they mainly exist in a compact conformation, 10% of K48 Ub chains exist in an open conformation exposing the hydrophobic patches to make these accessible for interactions with proteins such as the UBA domain of hHR23A which leads to the recruitment of K48 poly-ubiquitinated substrates for proteasomal degradation (Varadan et al., 2005). In another study, the K48 diUb molecule has been found to exist predominantly in an open conformation (Hirano et al., 2011). It is clear that the existence of multiple conformations of K48 polyUb chains in cells are essential to bind with different proteins and elicit different responses and further research is needed to study the structural dynamics of K48 polyUb chains in cells. Although X-ray data can reveal different conformations of diUb molecules, solution NMR is convenient to study the dynamics between different conformations and interactions with specific binding domains. Moreover, control of the environment in NMR experiments offers freedom to study solution structures at different physiological conditions, pH or temperature. Given the advances in chemical synthesis of Ub and Ub molecules containing thiolysine, we were able to generate distally labeled diUbs and studied the interactions between the two Ub moieties from the perspective of distal Ub. The synthesis of Ub chains by genetic incorporation of protected lysine residues using modified tRNA synthetases followed by selective chemical ligation and deprotection has also enabled generating diUb molecules of all linkages which were then analyzed by NMR spectroscopy (Castañeda et al., 2016b). Both approaches have demonstrated the advantages of using chemoenzymatic procedures to make diUb molecules to study their structural dynamics related to functionality.

For a better understanding of ubiquitin signaling pathway, it is essential to know how polyUb-specific interacting proteins recognize different polyUb chains. These interacting proteins often contain a specific UBD that can bind to specific polyUb chains, leading to different cellular responses. The best-studied Ub-interaction system is the K48 polyUb chain type and its corresponding interacting protein hHR23a in the proteasomal degradation system. Recently, it has been shown that hHR23a protein also recognizes K27 Ub chains, thereby implicating it in the DNA repair mechanism (Castañeda et al., 2016b). Although K48 chains are readily available for *in-vitro* studies, K27 chains are impossible to make via biochemical strategies and recombinant enzymes. Hence the chemical synthesis of these chains, such as shown in this study, may develop into a valuable tool in identifying the interacting proteins and establish a mechanism of binding.

DNA repair pathways are essential for the maintenance of the integrity of genomic DNA. The DNA repair pathway requires the efficient action of different protein complexes including the BRCA complex. Ubiquitination also plays an essential role in this pathway by adding different ubiquitin chains onto the proteins involved. For instance, the BRCA/ABRAXIS protein complex can be polyubiquitinated with K6, K48, and K63 polyUb chains by different sets of ubiquitin ligation enzymes and each of these modifications leads to different responses in the cell. Of special interest is the polyubiquitination with K6 chains which leads to recruitment of the DNA polymerase complex to restart DNA synthesis after DNA repair has been accomplished (Morris and Solomon, 2004). For K6 polyUb chains, UBXN1 acts as a specific receptor protein and its UBA domain has been reported to be involved in chain recognition. However, the exact mode of binding has not been shown using any biophysical methods so far. In this study, we showed that to achieve binding to K6-linked ubiquitin, instead of the canonical UBXN1 UBA (1-42) domain, an extended version of the UBXN1 UBA domain, UBA(ext1-52), is needed. For the first time, we gain structural insight into the recognition of this elusive K6-specific ubiquitin-binding domain. Our results suggest that different conformations of K6 chains are locked into one dominant conformation upon binding with the UBXN1 UBA(ext1-52) domain. The additional 10 amino acids long C-terminal extension of the conventional UBA domain is found to be conserved among different proteins and is therefore important to study this in more detail in future experiments.

## Conclusion

We have synthesized all isopeptide-linked distally ^15^N labeled diUb chains using native chemical ligation. This allowed us to study their conformations in solution and the interactions of the distal Ub moiety with the proximal Ub moiety by NMR. We also established that the additional C-terminal residues of the conventional UBA domain of UBXN1 protein are essential in binding specifically with K6 diUb molecule.

**Figure 8:**
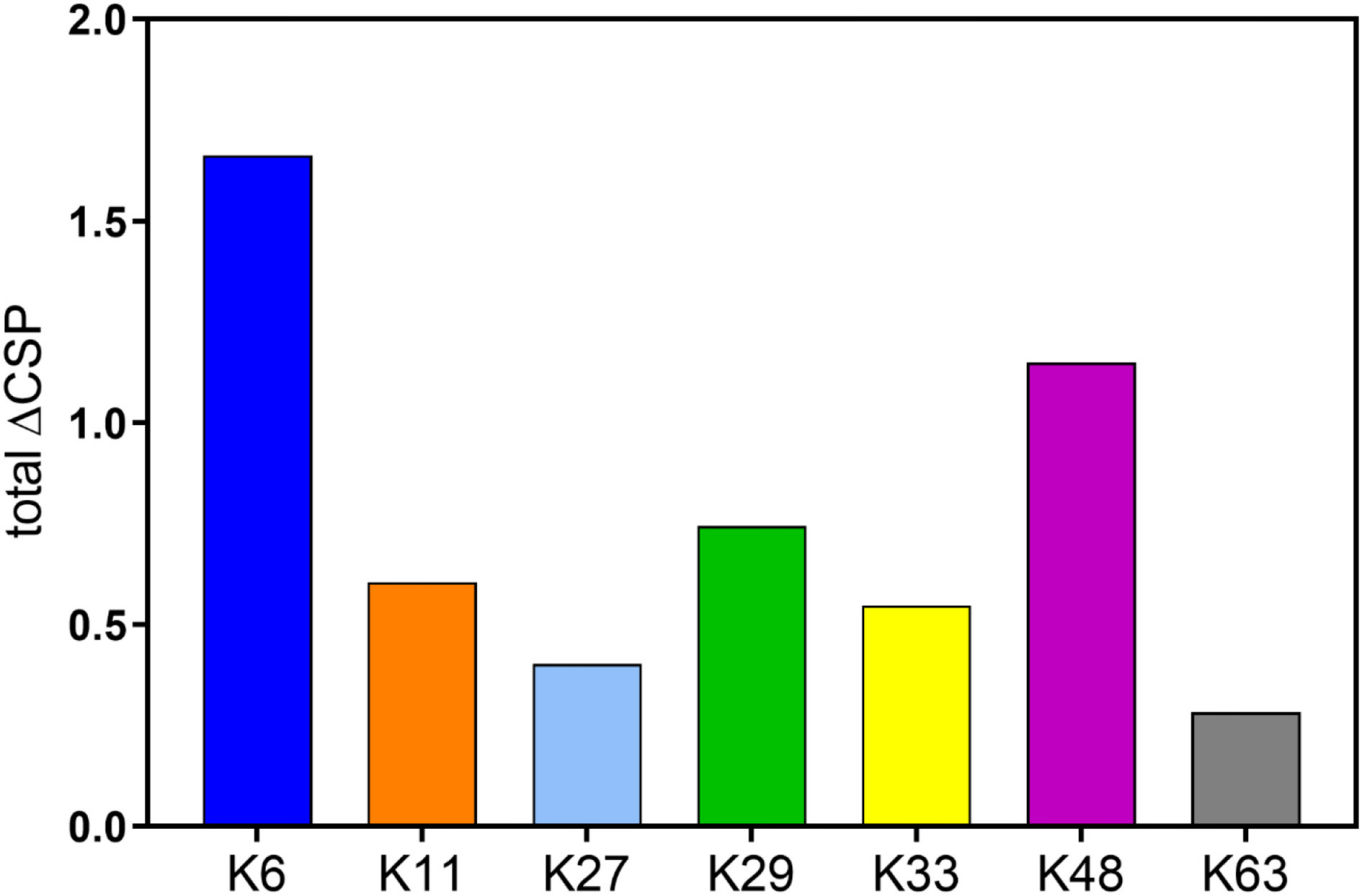
Sum of CSPs of residues in distal Ub of all diUbs excluding the C-terminal tail interactions which happen due to the proximity of isopeptide bond, and not exclusively due to the interaction between the interface of the distal and proximal Ub.

Upon comparing different diubiquitins of each linkage, we observed that K48-, K6-, K29-, and K11- diUbs were in a relatively closed conformation while K33-, K27-, and K63- diUbs were in a more open conformation. The CSPs revealed that K6 diUb exhibits the most closed conformation among all diubiquitins, whereas K63 exhibits the most open conformation. In general, calculating the total CSPs of all residues in each of the diUb spectra, excluding the C-terminal tail encompassing residues 70 to 76, provided a tentative overview on the degree of compactness for each of the diUb molecules (Figure 8). In addition, we found that certain diUbs like K6 diUb, K48 diUb, and K63 diUb exist in more than one conformation. For instance, in K6 diUb the residues Val5, Thr12, Ile13, Thr14, Asp32, and Ile36 gave rise to two signals.

Using our synthetic ^15^N diUbs, we established how only an extended version of the UBA domain (UBAext1-52) of the UBXN1 protein binds selectively to K6 diUb, using NMR titration experiments, revealing the crucial residues in the distal Ub of K6 diUb important for this interaction. With this, we demonstrate the applicability of these ^15^N labeled diUb chains as tools for gaining structural insights into the selective recognition of a unique UBD for a diUb linkage.

## Data Availability Statement

The datasets generated for this study are available on request to the corresponding author.

## Author Contributions

DS and RM prepared the Ub and diUb reagents for NMR measurements. GT did the FP and MST measurements for UBA(ext1-52) and diUb interactions. HW measured the NMR spectra. DS and HW analyzed the NMR data. DF, FE, and KH provided valuable suggestions and ideas. RB and HO supported the work with grants from NWO.

### Conflict of Interest

FE and HO declare competing financial interests as shareholder of UbiQ Bio BV. The remaining authors declare that the research was conducted in the absence of any commercial or financial relationships that could be construed as a potential conflict of interest.
