# Supplementary material for: Diubiquitin-Based NMR Analysis: Interactions Between Lys6-Linked diUb and UBA Domain of UBXN1
Source: Front Chem. 2020 Jan 22;7:921. doi: 10.3389/fchem.2019.00921 (PMC6987245; doi:10.3389/fchem.2019.00921)
Supplement: Supplementary file 1 [file Data_Sheet_1.PDF]

frontiers

# *Supplementary Material*

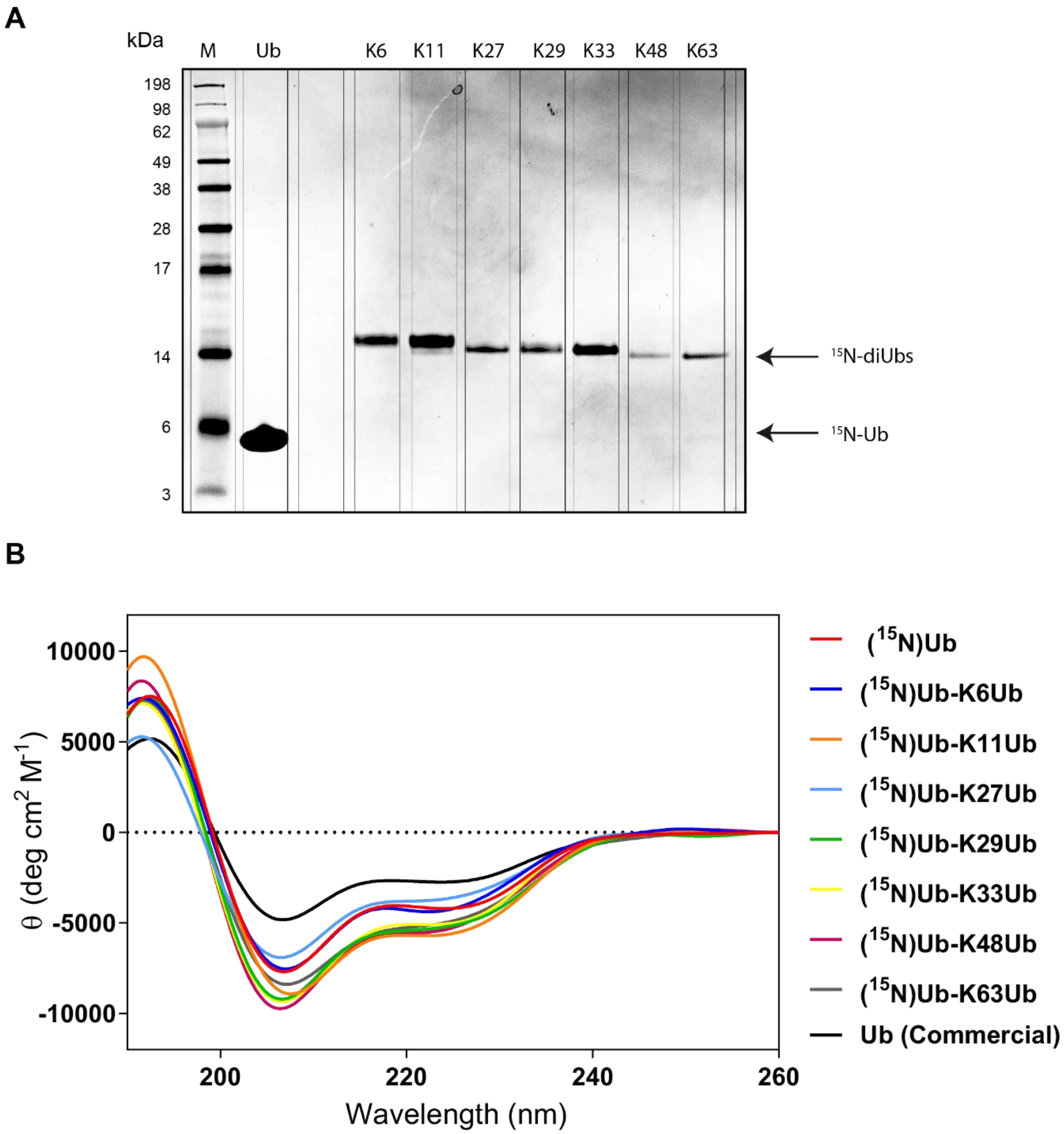


**Supplementary figure S1**: A) SDS-PAGE analysis of purified NMR samples. $^{15}$N-Ub was compared with $^{15}$N-diUbs which shows almost no contamination with monoUb samples. B) Circular Dichroism spectrum of $^{15}$N-Ub and $^{15}$N-diUbs compared with expressed Ub from a commercial source (Boston Biochem CAT: U-100H).

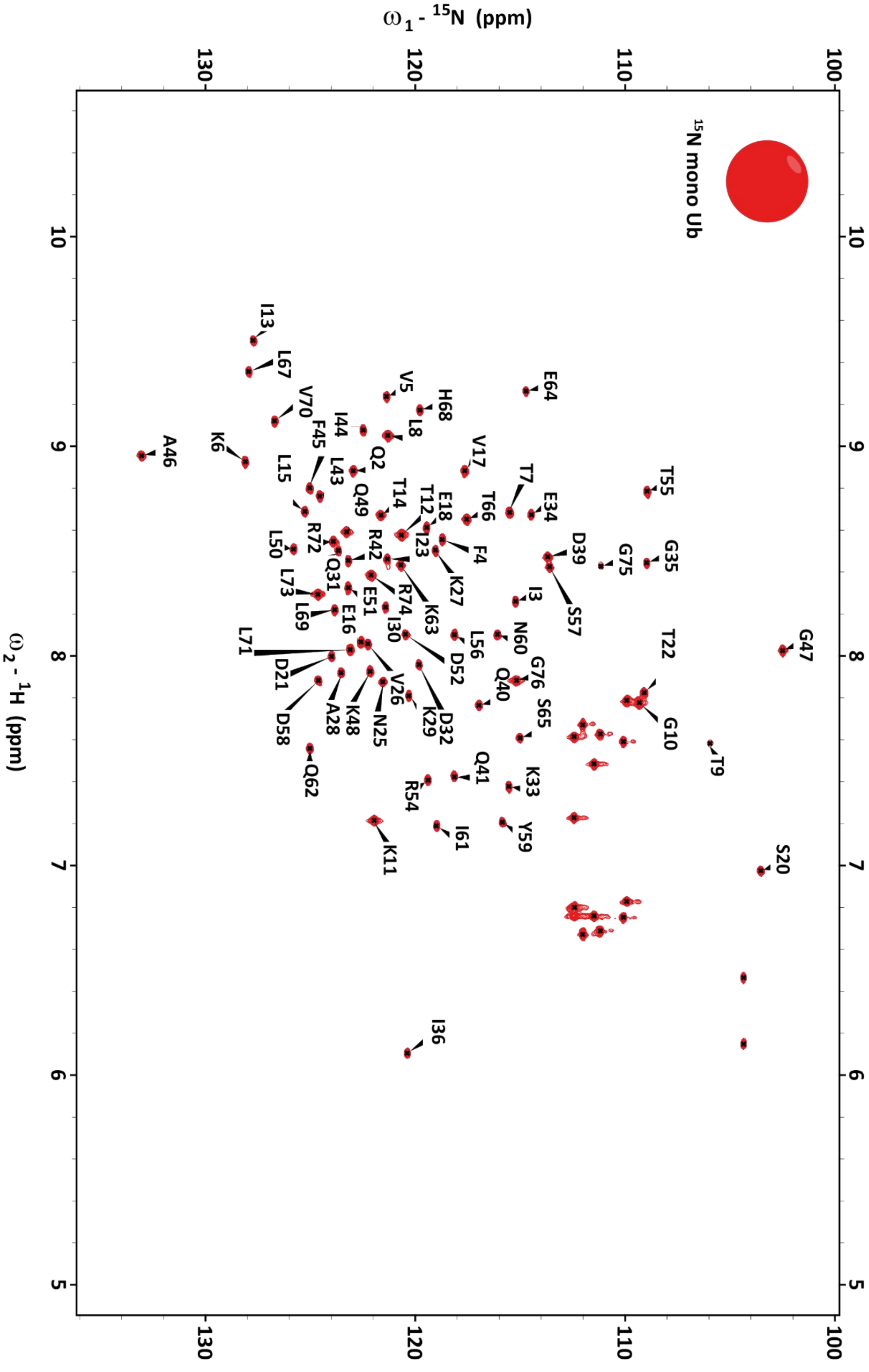


**Supplementary figure S2**: 2D NMR spectrum of $^{15}$N-Ub. The NMR ($^{1}$H-$^{15}$N HSQC) spectrum of free Ub (red) shows the assignments of main chain $^{1}$H-$^{15}$N cross peaks of almost all the residues.

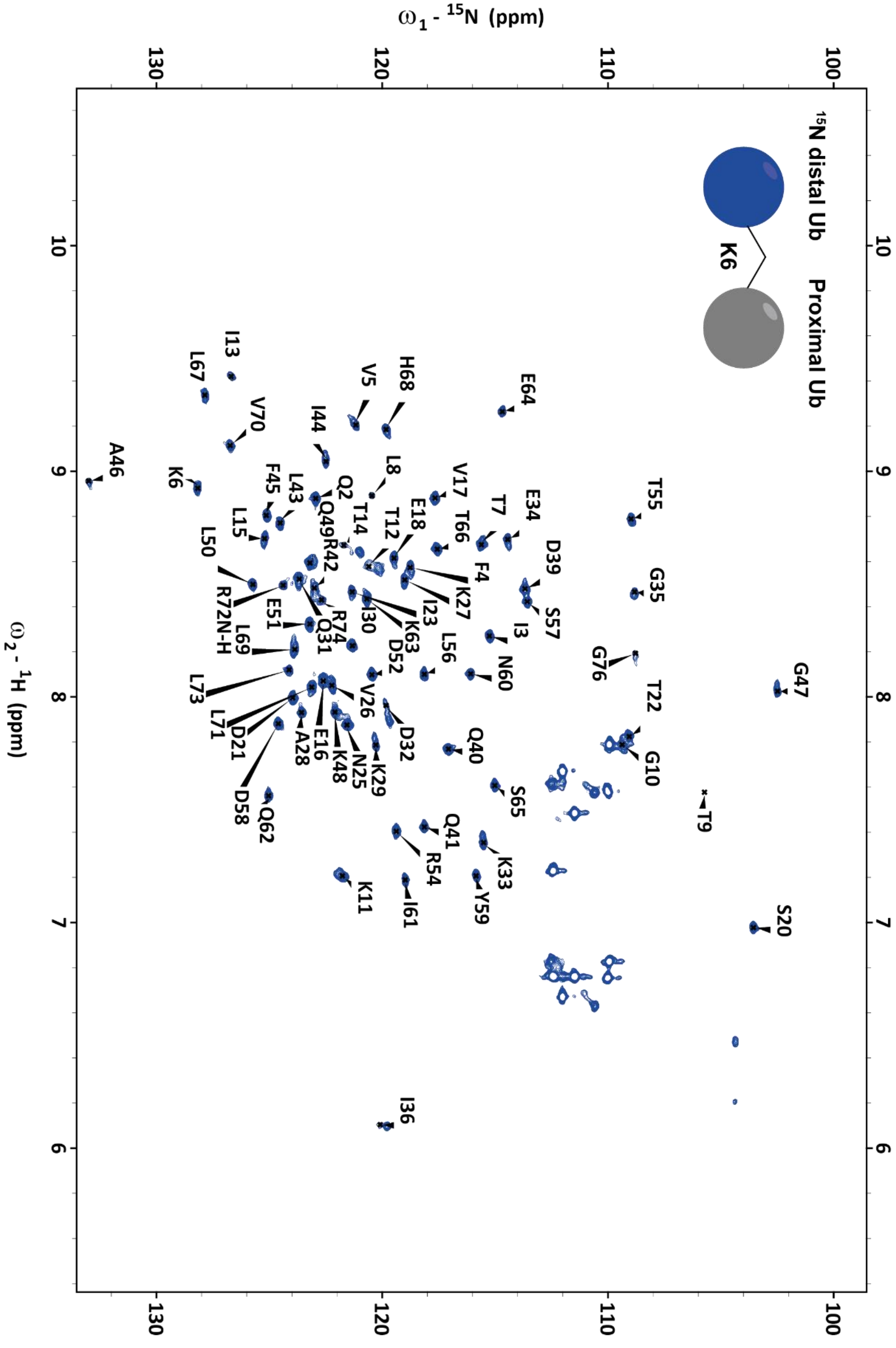


**Supplementary figure S3**: 2D NMR spectrum of $^{15}$N-Ub-K6-Ub (K6diUb). The NMR ($^{1}$H-$^{15}$N HSQC) spectrum of $^{15}$N-labelled distal Ub (blue) in a K6 diUb molecule shows displacement of some residues as quantified in Figure 3A.

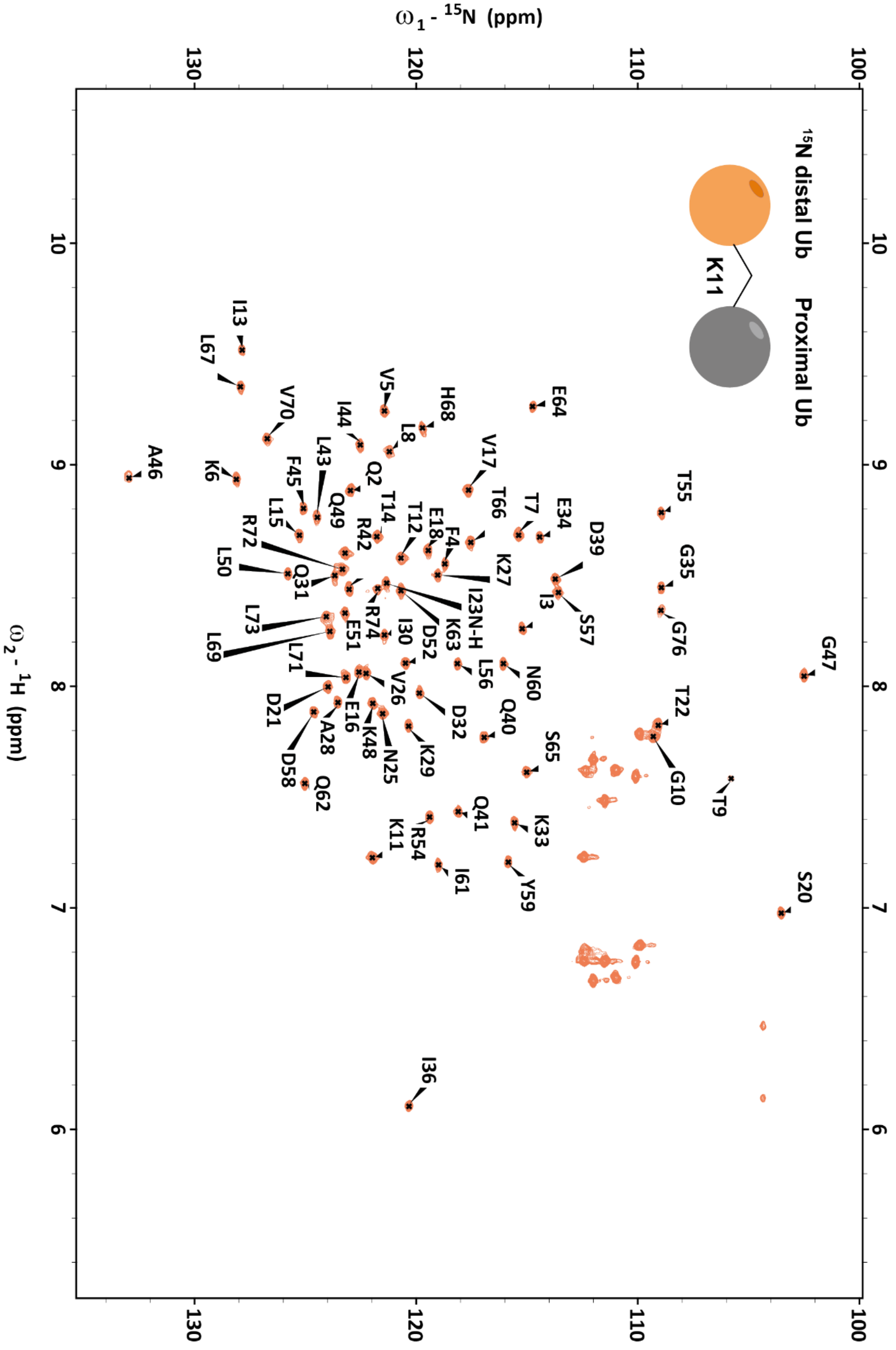


**Supplementary figure S4**: 2D NMR spectrum of $^{15}N$-Ub-K11-Ub (K11 diUb). The NMR ($^{1}H$-$^{15}N$ HSQC) spectrum of $^{15}N$-labelled distal Ub (orange) in a K11 diUb molecule shows displacement of some residues as quantified in Figure 3B.

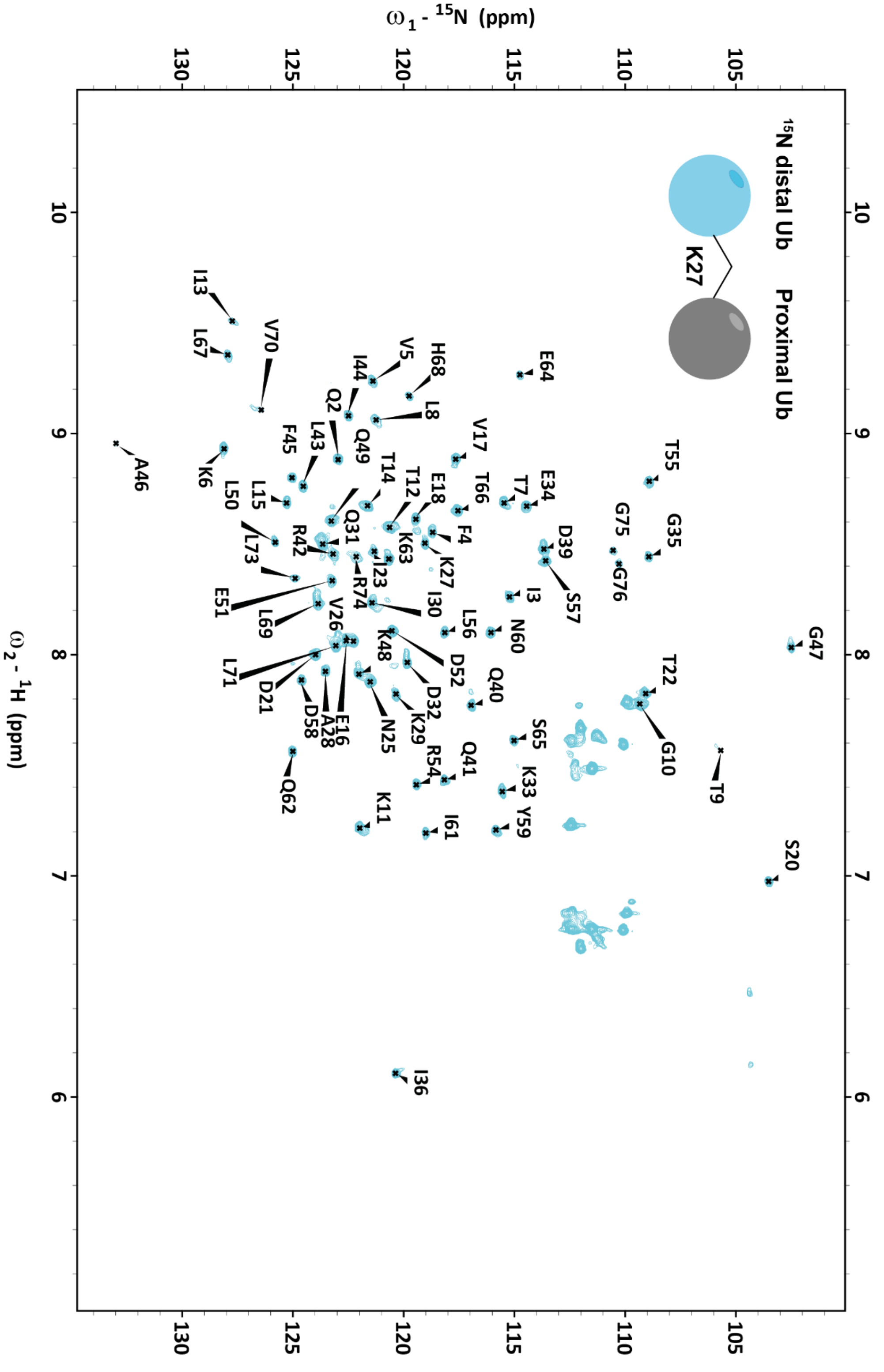


**Supplementary figure S5**: NMR spectrum of $^{15}$N-Ub-K27-Ub (K27 diUb). The NMR ($^{1}$H-$^{15}$N HSQC) spectrum of $^{15}$N-labelled distal Ub (light blue) in a K27 diUb molecule shows displacement of some residues as quantified in Figure 3C.

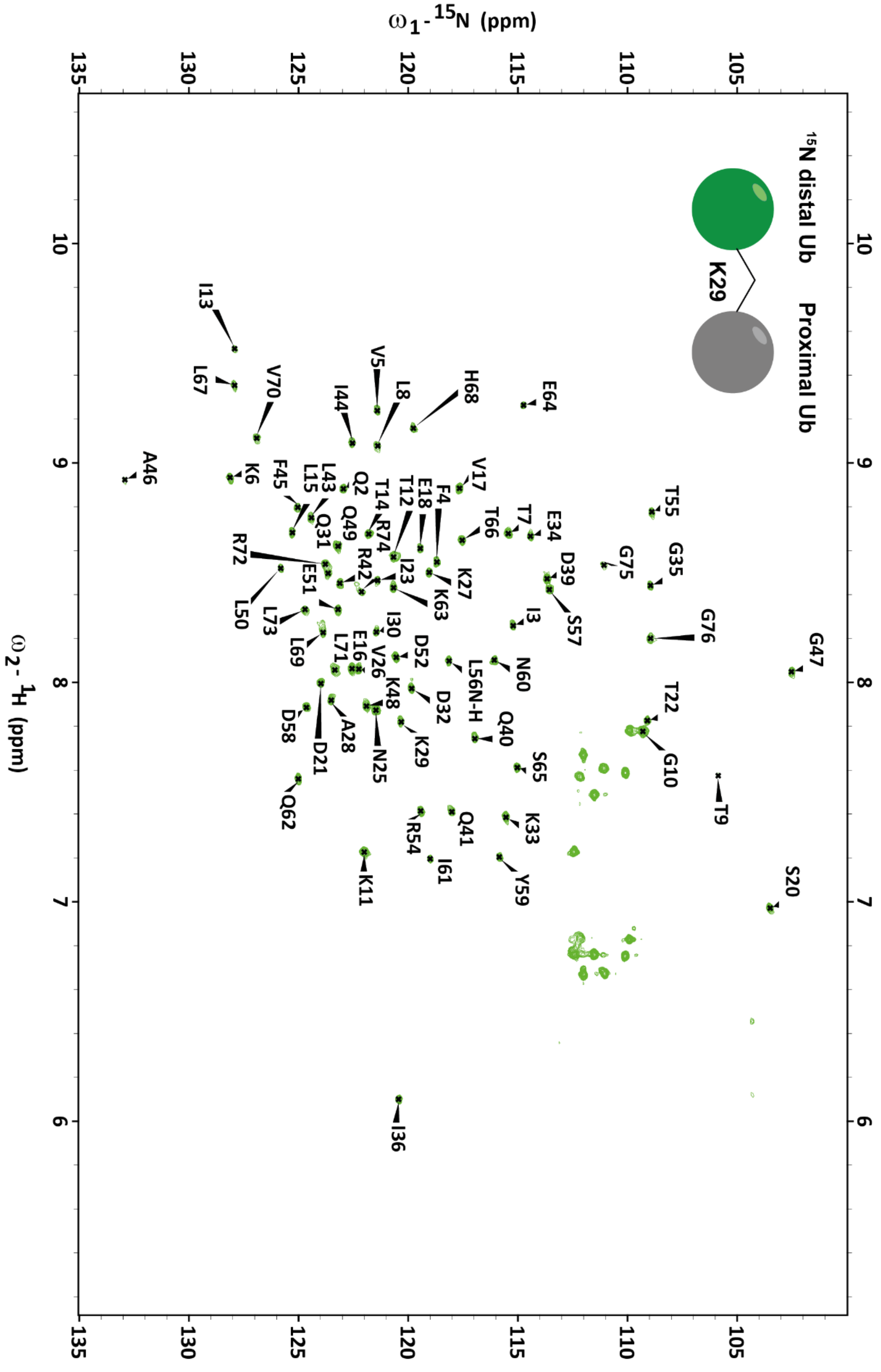


**Supplementary figure S6**: NMR spectrum of $^{15}$N-Ub-K29-Ub (K29 diUb). The NMR ($^{1}$H-$^{15}$N HSQC) spectrum of $^{15}$N-labelled distal Ub (green) in a K29 diUb molecule shows displacement of some residues as quantified in Figure 3D.

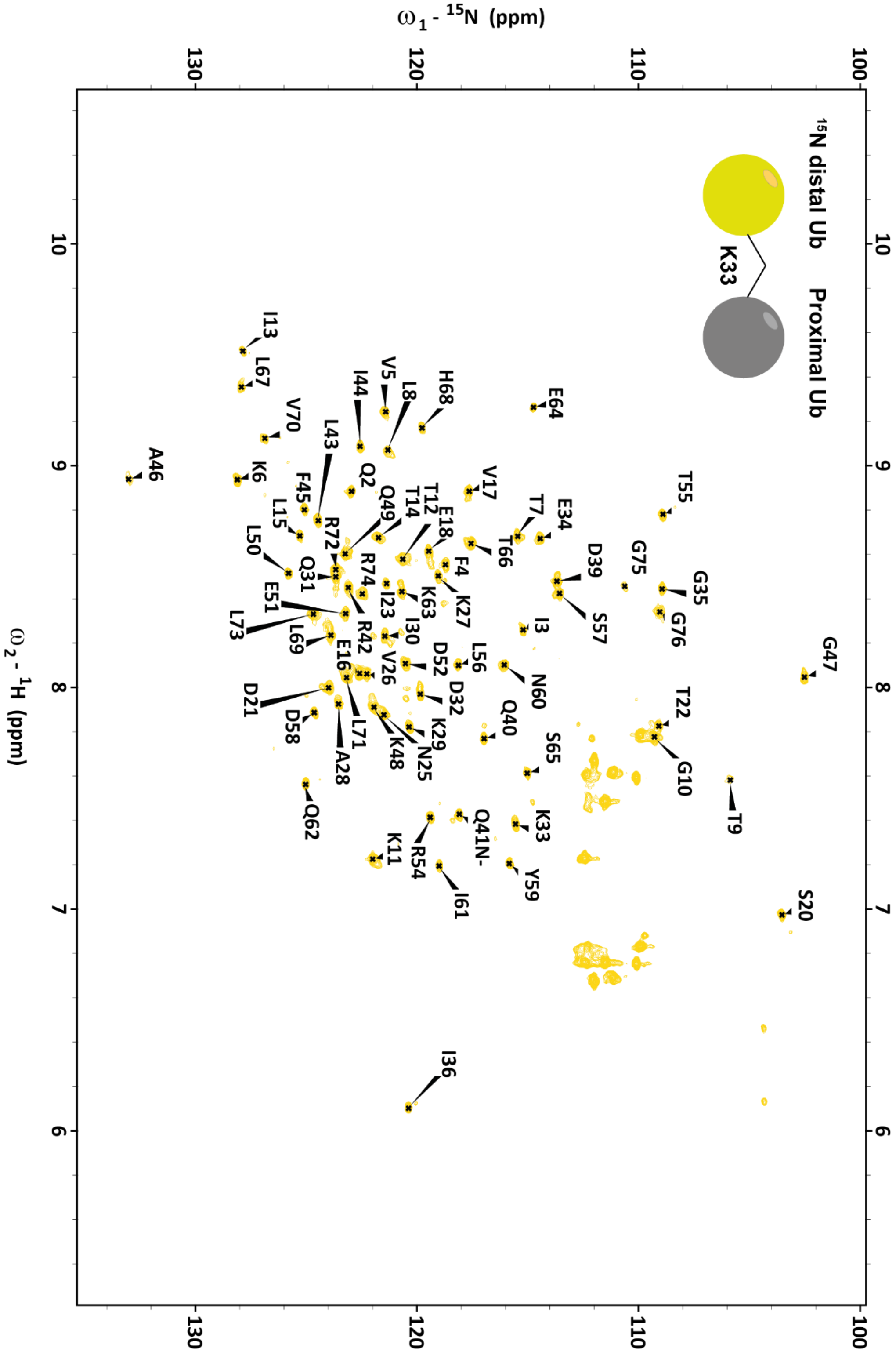


**Supplementary figure S7**: NMR spectrum of $^{15}$N-Ub-K33-Ub (K33 diUb). The NMR ($^{1}$H-$^{15}$N HSQC) spectrum of $^{15}$N-labelled distal Ub (yellow) in a K33 diUb molecule shows displacement of some residues as quantified in Figure 3E.

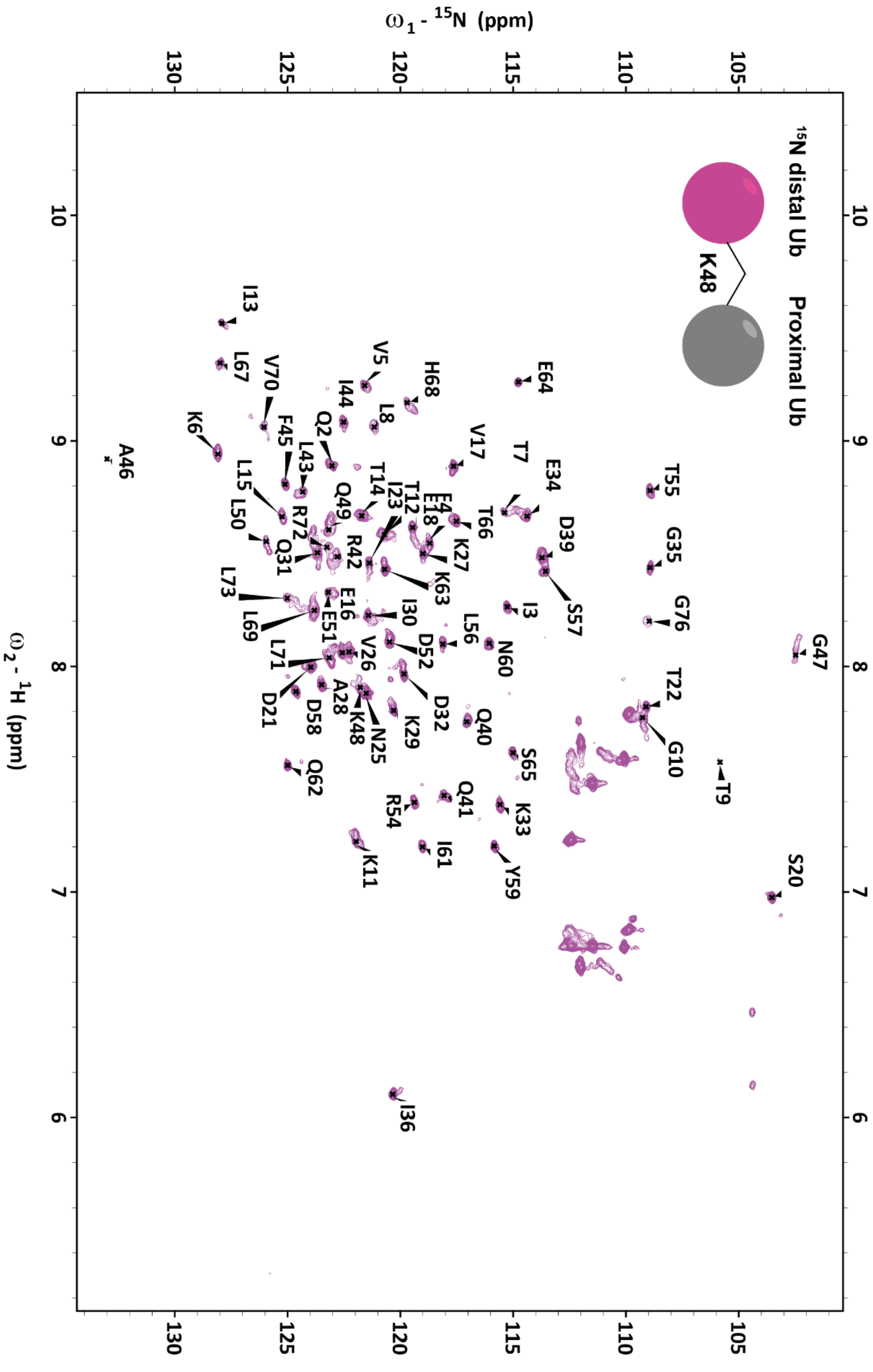


**Supplementary figure S8**: NMR spectrum of $^{15}$N-Ub-K48-Ub (K48 diUb). The NMR ($^{1}$H-$^{15}$N HSQC) spectrum of $^{15}$N-labelled distal Ub (magenta) in a K48 diUb molecule shows displacement of some residues as quantified in Figure 3F.

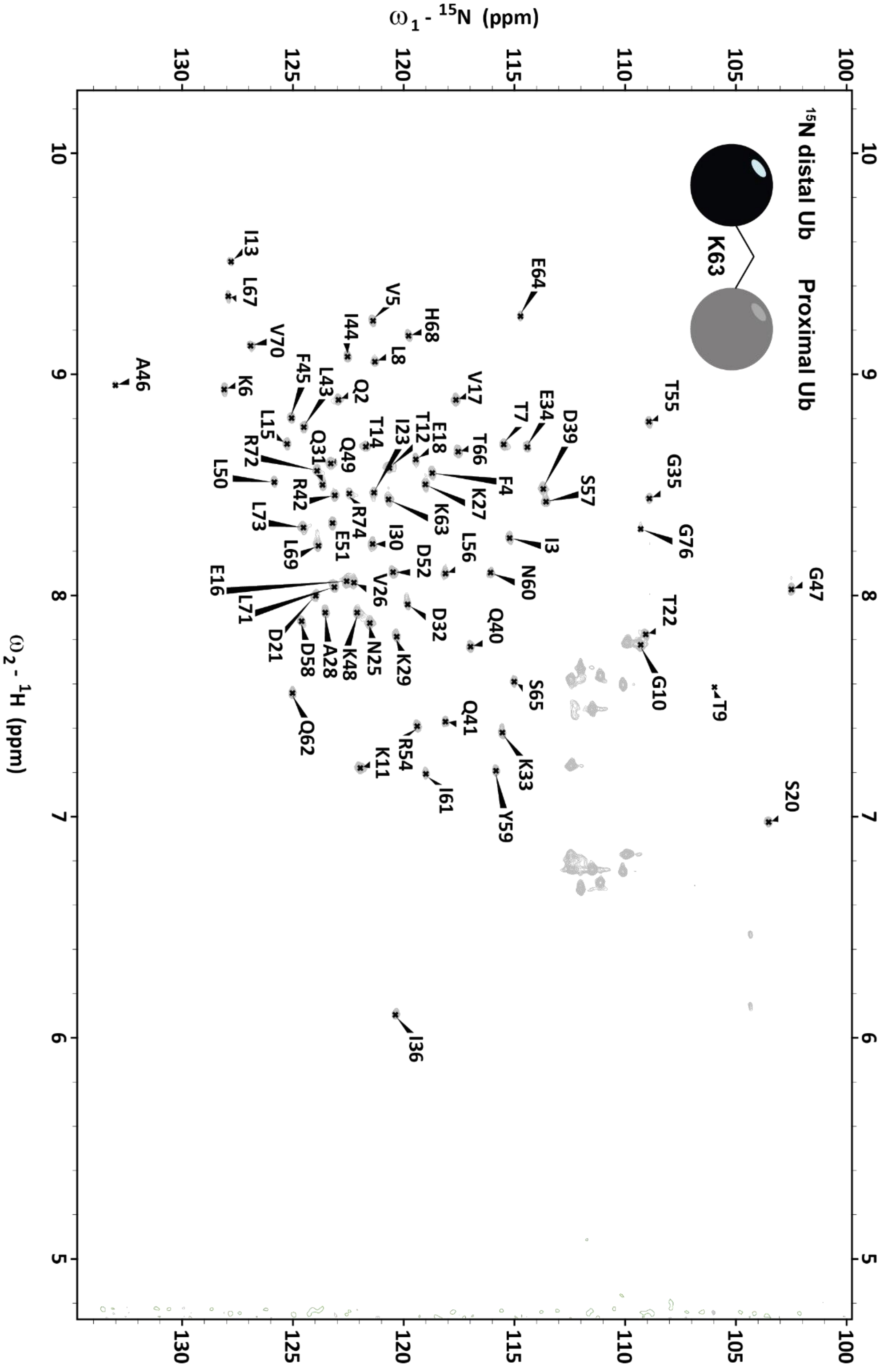


**Supplementary figure S9**: NMR spectrum of $^{15}$N-Ub-K63-Ub (K63 diUb). The NMR ($^1$H-$^{15}$N HSQC) spectrum of $^{15}$N-labelled distal Ub (grey) in a K63 diUb molecule shows displacement of some residues as quantified in Figure 3G.

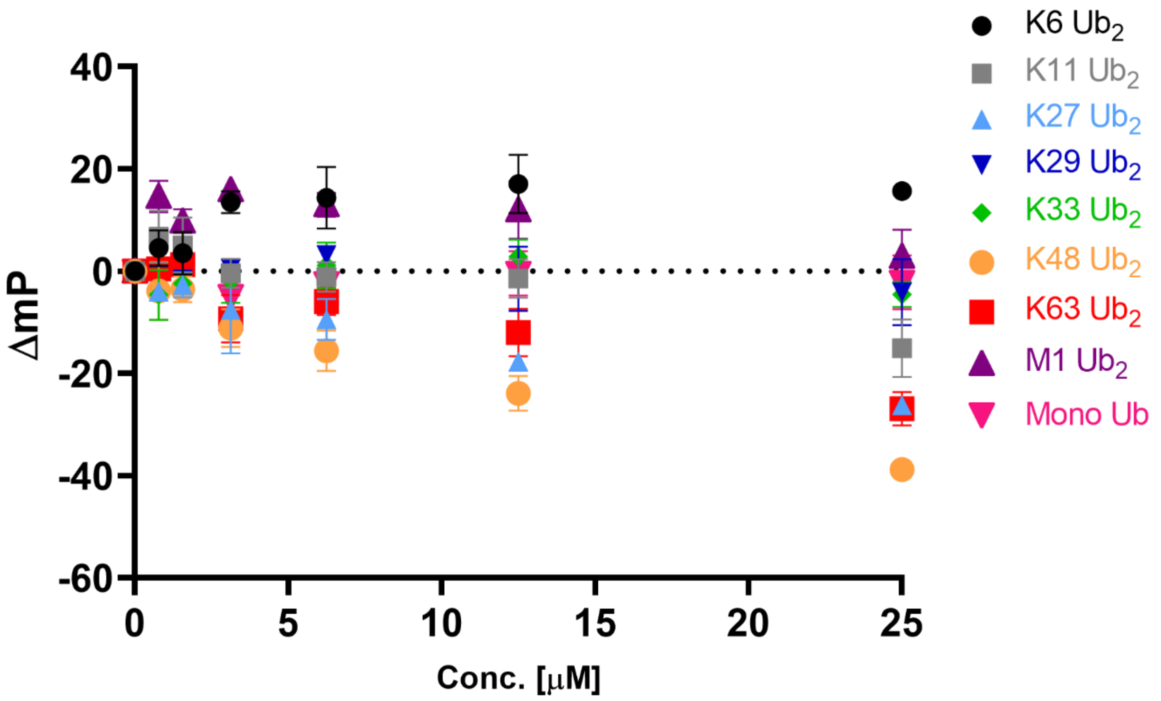


**Supplementary figure S10**: Fluorescence polarization assay using TAMRA-labeled UBXN1 UBA (1-42) domain and different concentrations of all 8 homotypical diUbs and monoUb. There were no interactions of this binding domain with any of the diUbs and the monoUb tested here.

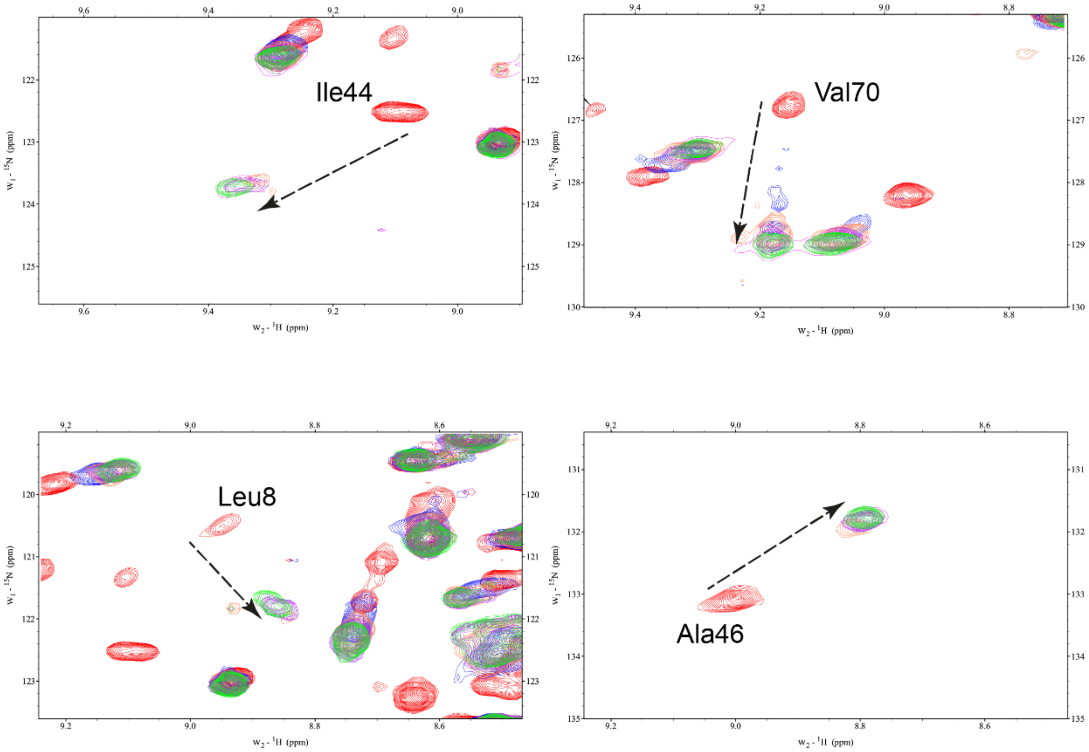


**Supplementary figure S11**: NMR spectrum showing key residues of the hydrophobic region in $^{15}$N-Ub-K6-Ub (K6 diUb) added with different concentrations of unlabeled UBA(ext1-52) domain of UBXN1. Different colors represent the NMR ($^{1}$H-$^{15}$N HSQC) spectrum of $^{15}$N-labelled distal Ub in a K6 diUb molecule before (red) and after addition of 108 µM (blue), 135 µM (coral), 175 µM (purple), 270 µM (magenta) and 570 µM (green) of UBA domain

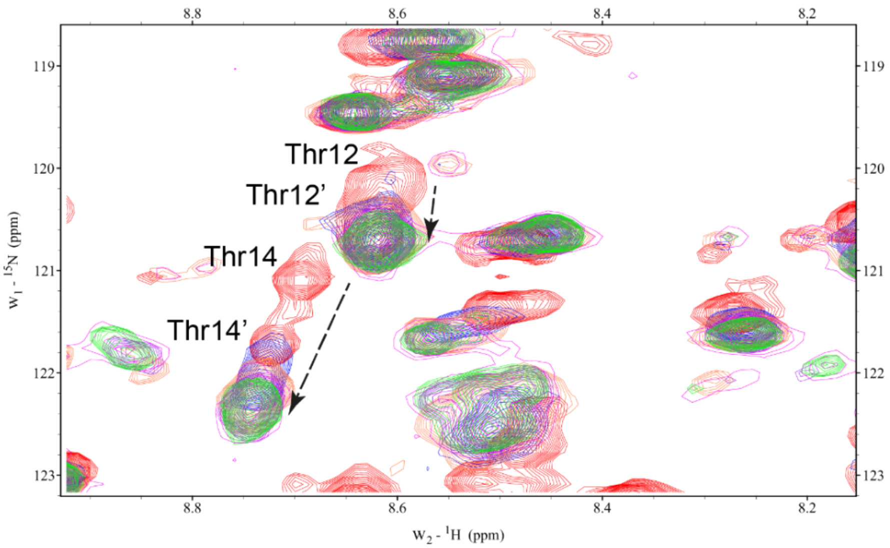


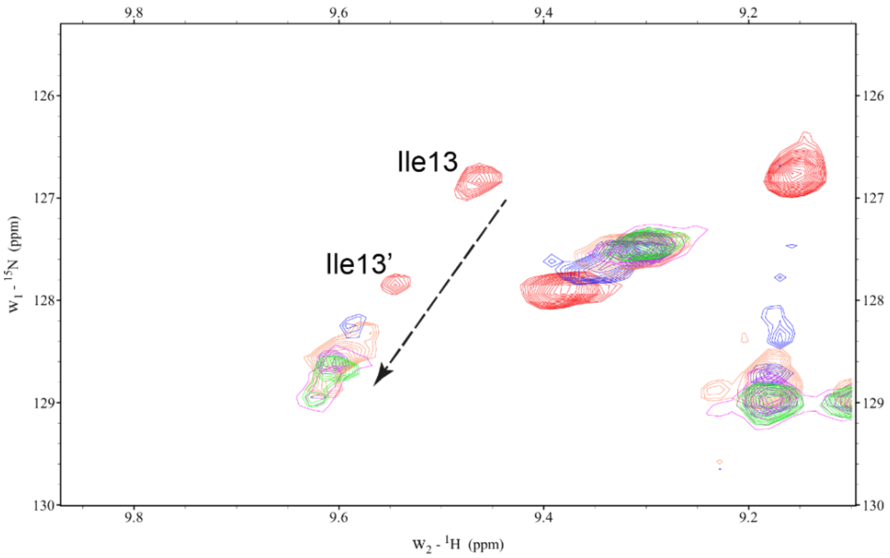


**Supplementary figure S12**: NMR spectrum showing Thr12, Thr14 (Top) and Ile13 (Bottom) signal shift in $^{15}$N-Ub-K6-Ub (K6 diUb) added with different concentrations of unlabeled UBA(ext1-52) domain of UBXN1. Different colors represent the NMR ($^{1}$H-$^{15}$N HSQC) spectrum of 15N-labelled distal Ub in a K6 diUb molecule before (red) and after addition of 108 µM (blue), 135 µM (coral), 175 µM (purple), 270 µM (magenta) and 570 µM (green) of UBA domain.

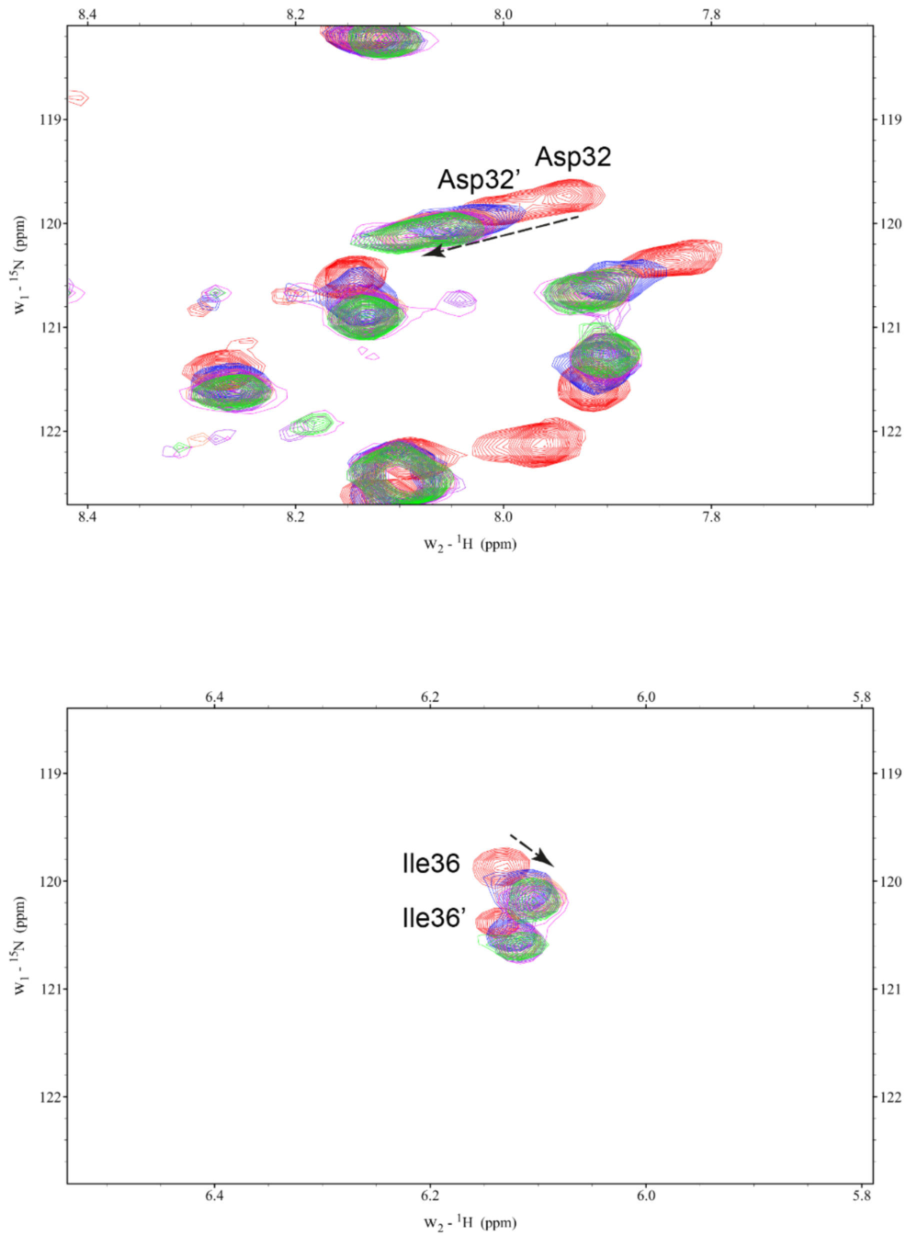


**Supplementary figure S13**: NMR spectrum showing split signals of Asp32 (Top) and Ile36 (Bottom) in $^{15}$N-Ub-K6-Ub (K6 diUb) added with different concentrations of unlabeled UBA(ext1-52) domain of UBXN1. The NMR ($^{1}$H-$^{15}$N HSQC) spectrum of 15N-labelled distal Ub in a K6 diUb molecule before (red) and after addition of 108 µM (blue), 135 µM (coral), 175 µM (purple), 270 µM (magenta) and 570 µM (green) of UBA domain.

$^{15}$N-Ub

$^{15}$N-MQIFVKTLTGKTITLEVEPSDTIENVKAKIQDKEGIPPDQQRLIFAGKQLEDGRTLSDYNIQKESTLHLVLRLRGG

Calculated MW: 8670 Da
Observed MW: 8669 Da

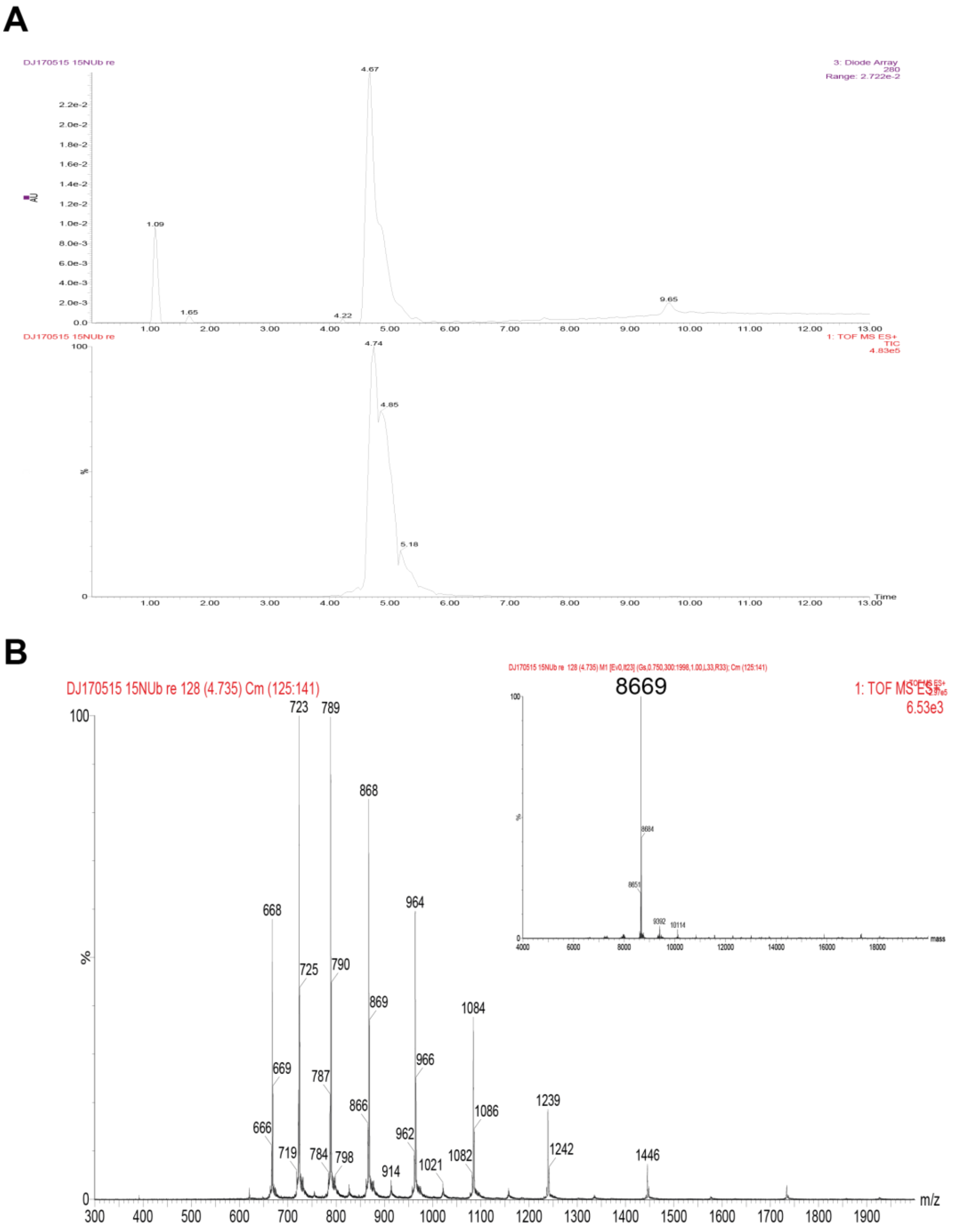


**Supplementary figure S14**: LC-MS analysis of $^{15}$N Ub sample. A. Top: UV chromatogram (λ – 280 nm); Bottom: Mass spectrum. B. Combined mass spectrum of peak at 4.67 min; Inset: Deconvoluted mass of mass spectra.

$^{15}$N-Ub-K6Ub

$^{15}$N-MQIFVKTLTGKTITLEVEPSDTIENVKAKIQDKEGIPPDQQRLIFAGKQLEDGRTLSDYNIQKESTLHLVLRLRGG

MQIFVKTLTGKTITLEVEPSDTIENVKAKIQDKEGIPPDQQRLIFAGKQLEDGRTLSDYNIQKESTLHLVLRLRGG

Calculated MW: 17216 Da
Observed MW: 17212 Da

**A**

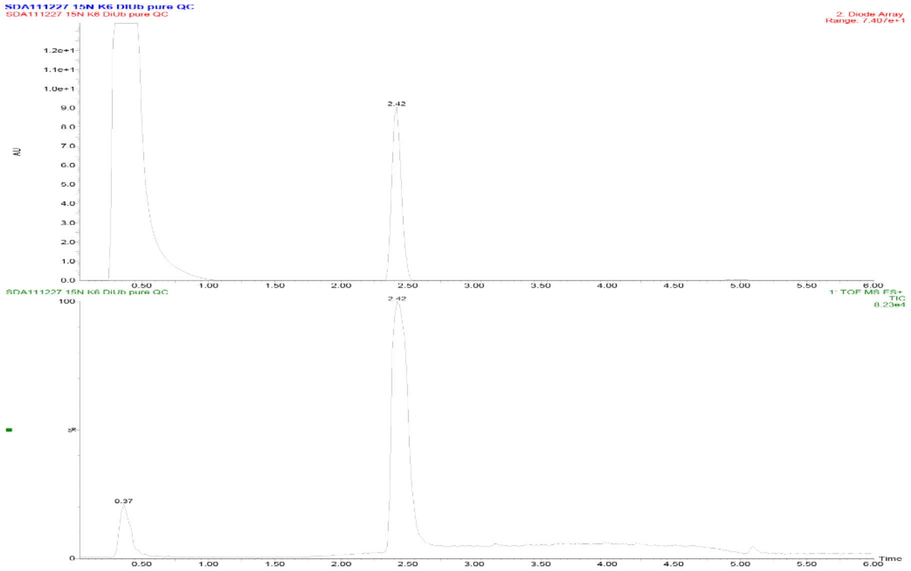


**B**

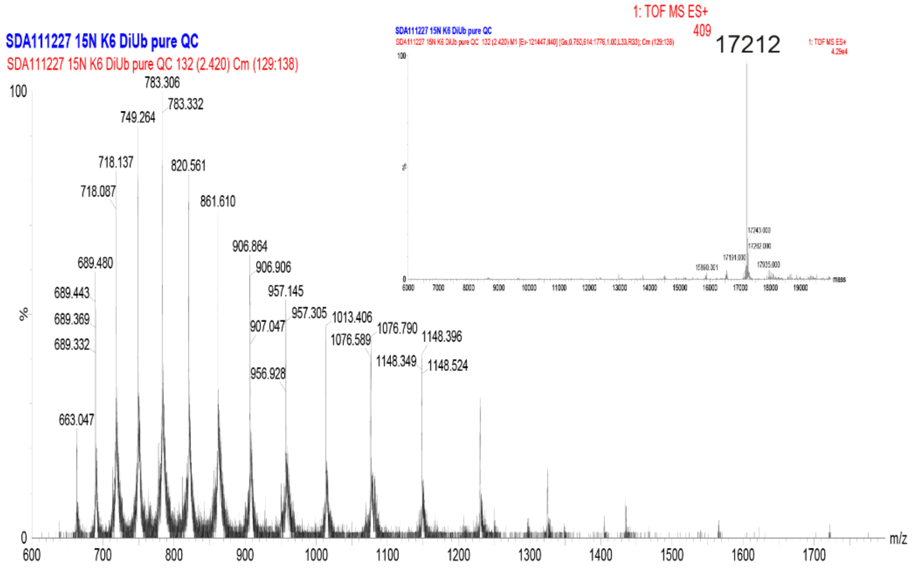


**Supplementary figure S15**: LC-MS analysis of $^{15}$N K6 diUb sample. A. Top: UV chromatogram (λ – 280 nm); Bottom: Mass spectrum. B. Combined mass spectrum of peak at 2.42 min; Inset: Deconvoluted mass of mass spectra.

$^{15}$N-Ub-K11Ub

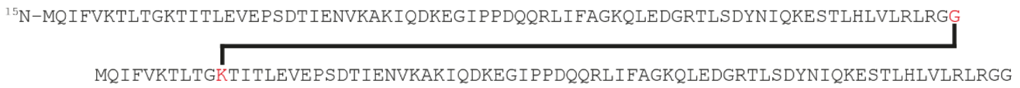


Calculated MW: 17216 Da
Observed MW: 17212 Da

**A**

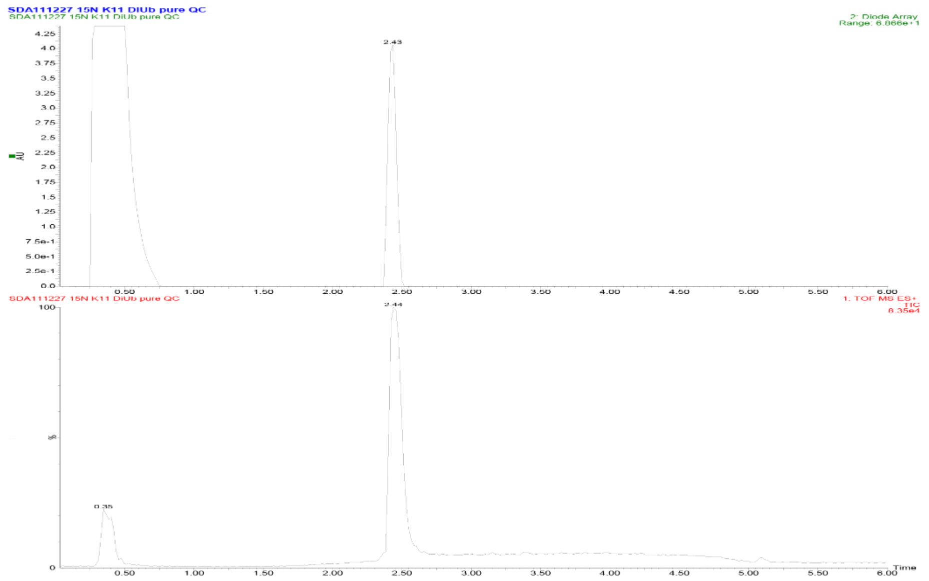


**B**

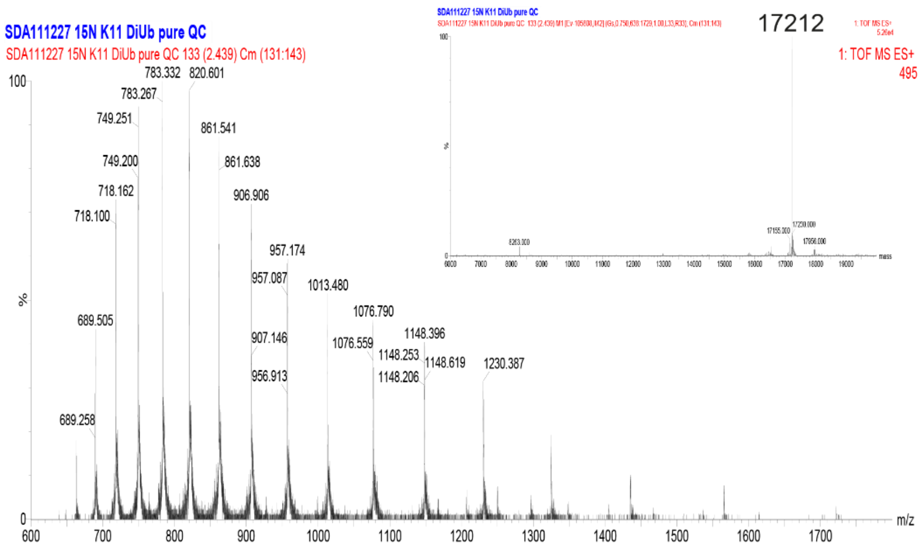


**Supplementary figure S16**: LC-MS analysis of $^{15}$N K11 diUb sample. A. Top: UV chromatogram (λ – 280 nm); Bottom: Mass spectrum. B. Combined mass spectrum of peak at 2.44 min; Inset: Deconvoluted mass of mass spectra.

$^{15}$N-Ub-K27Ub

$^{15}$N-MQIFVKTLTGKTITLEVEPSDTIENVKAKIQDKEGIPPDQQRLIFAGKQLEDGRTLSDYNIQKESTLHLVLRLRGG

MQIFVKTLTGKTITLEVEPSDTIENVKAKIQDKEGIPPDQQRLIFAGKQLEDGRTLSDYNIQKESTLHLVLRLRGG

Calculated MW: 17216 Da
Observed MW: 17212 Da

A

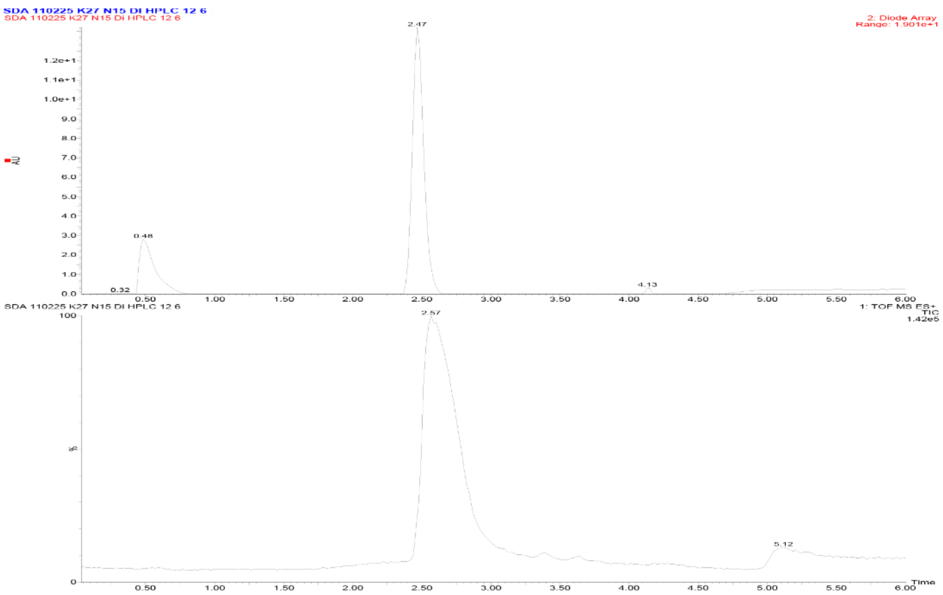


B

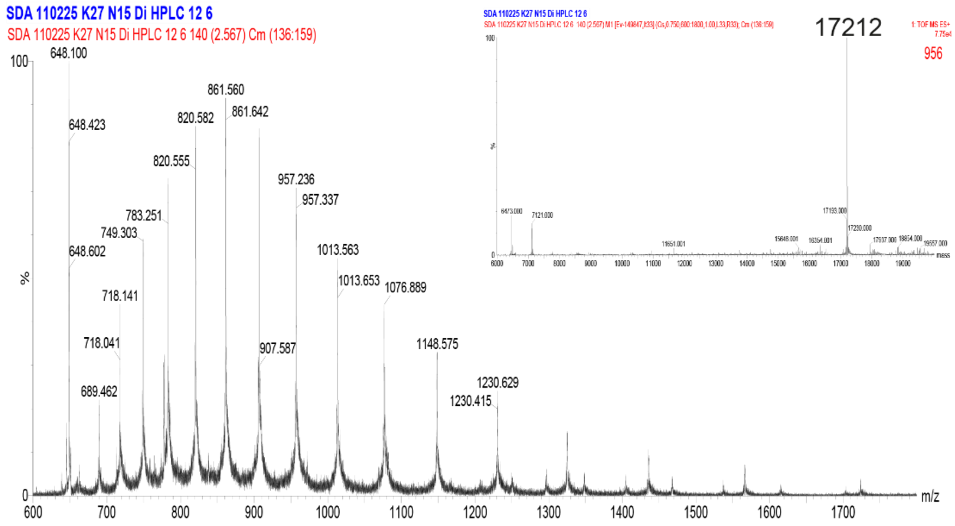


**Supplementary figure S17**: LC-MS analysis of $^{15}$N K27 diUb sample. A. Top: UV chromatogram ($\lambda$ – 280 nm); Bottom: Mass spectrum. B. Combined mass spectrum of peak at 2.47 min; Inset: Deconvoluted mass of mass spectra.

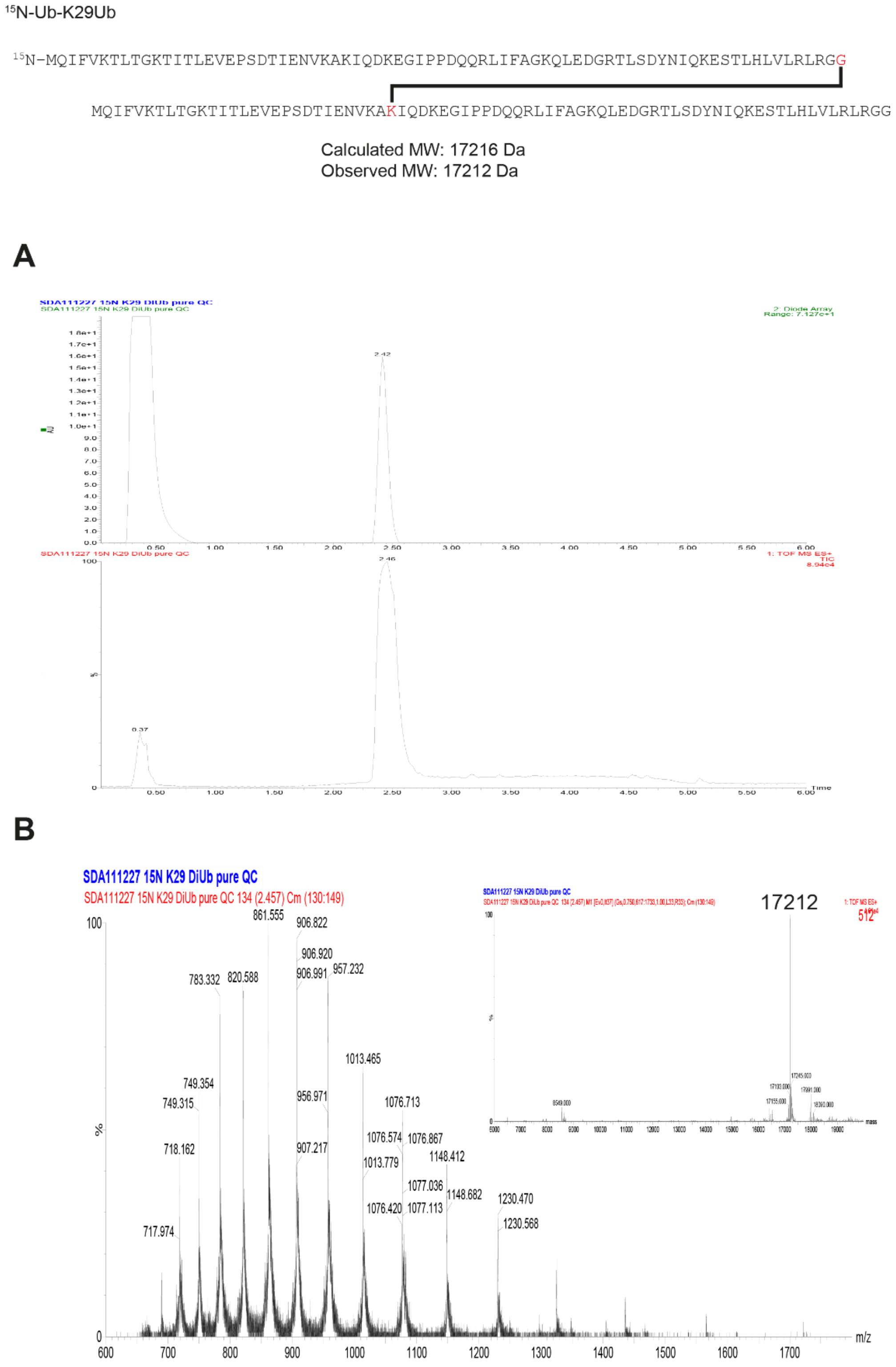


**Supplementary figure S18**: LC-MS analysis of $^{15}$N K29 diUb sample. A. Top: UV chromatogram (λ – 280 nm); Bottom: Mass Spectrum. B. Combined mass spectrum of peak at 2.46 min; Inset: Deconvoluted mass of mass spectra.

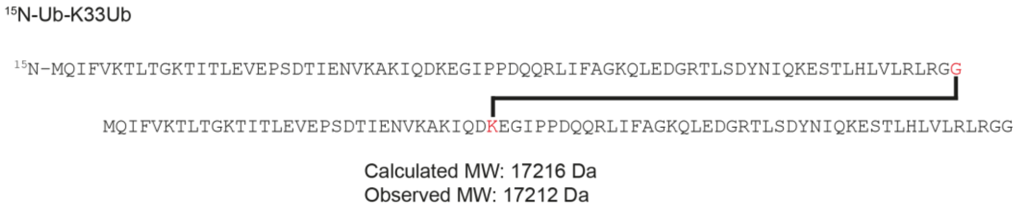


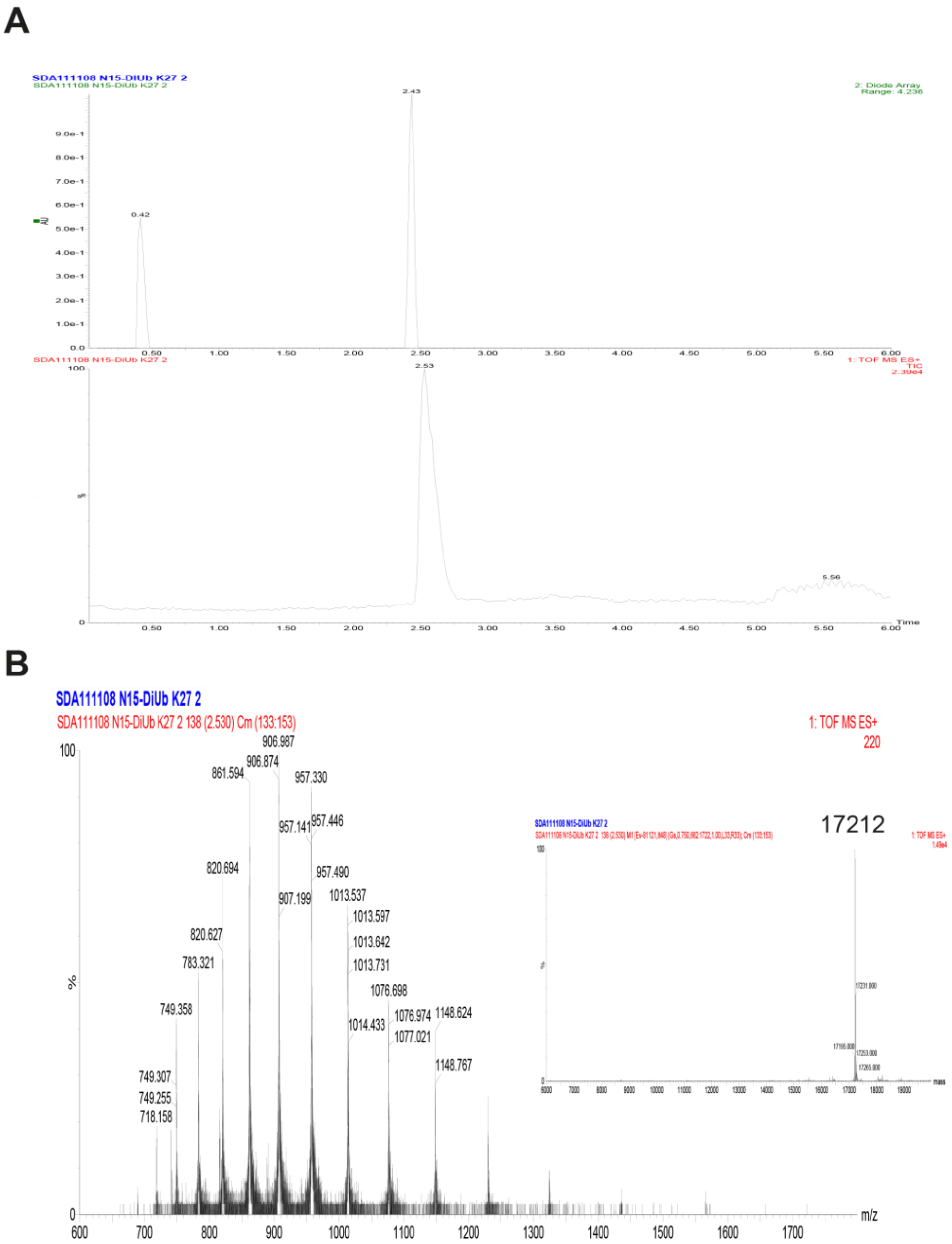


**Supplementary figure S19**: LC-MS analysis of $^{15}$N K33 diUb sample. A. Top: UV chromatogram (λ – 280 nm); Bottom: Mass spectrum. B. Combined mass spectrum of peak at 2.43 min; Inset: Deconvoluted mass of mass spectra.

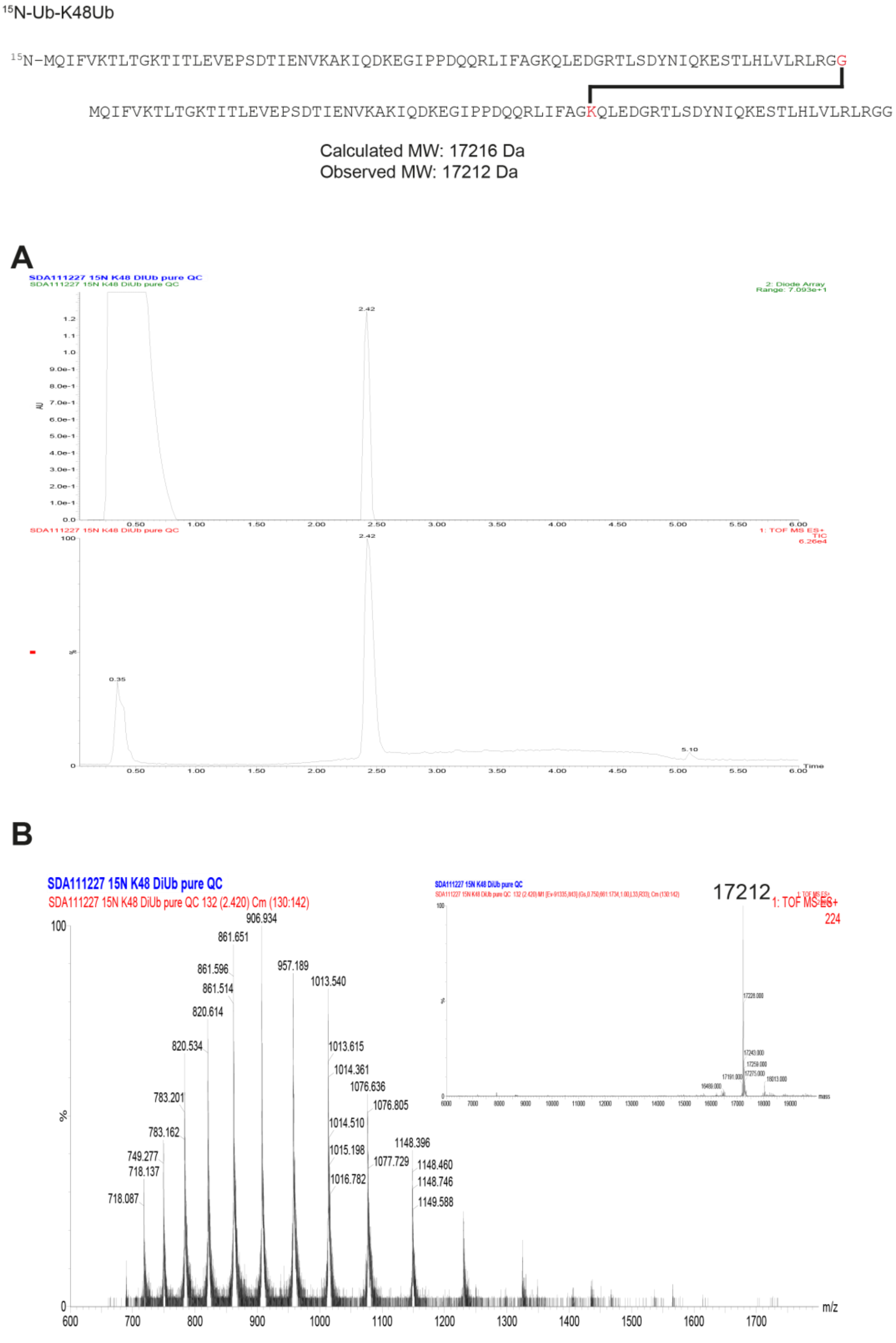


**Supplementary figure S20**: LC-MS analysis of $^{15}$N K48 diUb sample. A. Top: UV chromatogram (λ – 280 nm); Bottom: Mass spectrum. B. Combined mass spectrum of peak at 2.42 min; Inset: Deconvoluted mass of mass spectra.

$^{15}$N-Ub-K63Ub

$^{15}$N-MQIFVKTLTGKTITLEVEPSDTIENVKAKIQDKEGIPPDQQRLIFAGKQLEDGRTLSDYNIQKESTLHLVLRLRGG

MQIFVKTLTGKTITLEVEPSDTIENVKAKIQDKEGIPPDQQRLIFAGKQLEDGRTLSDYNIQKESTLHLVLRLRGG

Calculated MW: 17216 Da
Observed MW: 17212 Da

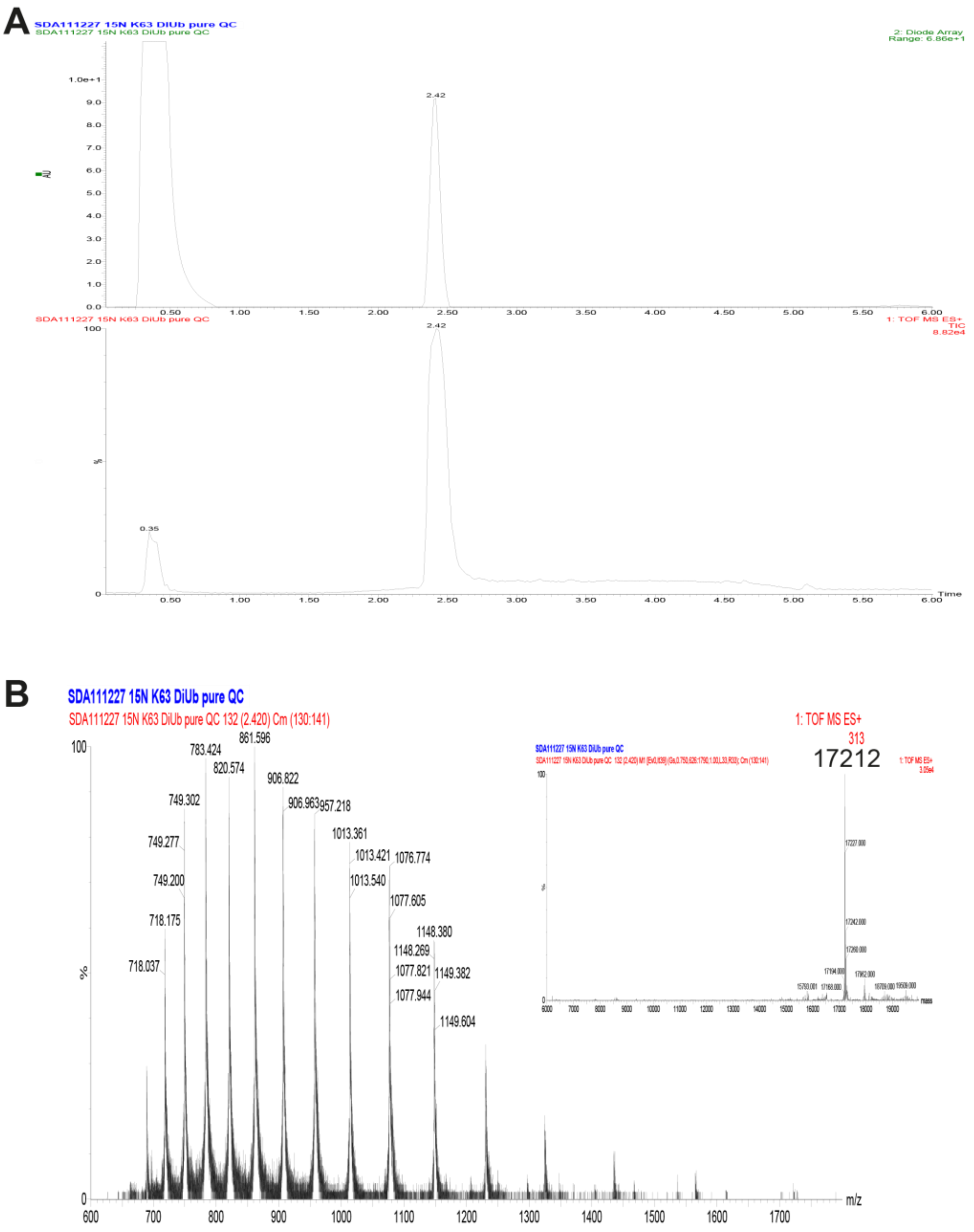


**Supplementary figure S21**: LC-MS analysis of $^{15}$N K63 diUb sample. A. Top: UV chromatogram (λ – 280 nm); Bottom: Mass spectrum. B. Combined mass spectrum of peak at 2.42 min; Inset: Deconvoluted mass of mass spectra.
